# The *de novo* Purine Biosynthesis Pathway Is the Only Commonly Regulated Cellular Pathway during Biofilm Formation in TSB-Based Medium in Staphylococcus aureus and Enterococcus faecalis

**DOI:** 10.1128/Spectrum.00804-21

**Published:** 2021-12-22

**Authors:** Martin Gélinas, Léa Museau, Arielle Milot, Pascale B. Beauregard

**Affiliations:** a Université de Sherbrooke, Département de biologie, Faculté des sciences, Sherbrooke, Québec, Canada; University of Calgary

**Keywords:** 29212, *Enterococcus faecalis*, *Staphylococcus aureus*, V583, biofilm, purin biosynthesis pathway, SH1000, transcriptomic, USA300

## Abstract

Bacterial biofilms are involved in chronic infections and confer 10 to 1,000 times more resistance to antibiotics compared with planktonic growth, leading to complications and treatment failure. When transitioning from a planktonic lifestyle to biofilms, some Gram-positive bacteria are likely to modulate several cellular pathways, including central carbon metabolism, biosynthesis pathways, and production of secondary metabolites. These metabolic adaptations might play a crucial role in biofilm formation by Gram-positive pathogens such as Staphylococcus aureus and Enterococcus faecalis. Here, we performed a transcriptomic approach to identify cellular pathways that might be similarly regulated during biofilm formation in these bacteria. Different strains and biofilm-inducing media were used to identify a set of regulated genes that are common and independent of the environment or accessory genomes analyzed. Our approach highlighted that the *de novo* purine biosynthesis pathway was upregulated in biofilms of both species when using a tryptone soy broth-based medium but not so when a brain heart infusion-based medium was used. We did not identify other pathways commonly regulated between both pathogens. Gene deletions and usage of a drug targeting a key enzyme showed the importance of this pathway in biofilm formation of S. aureus. The importance of the *de novo* purine biosynthesis pathway might reflect an important need for purine during biofilm establishment, and thus could constitute a promising drug target.

**IMPORTANCE** Biofilms are often involved in nosocomial infections and can cause serious chronic infections if not treated properly. Current anti-biofilm strategies rely on antibiotic usage, but they have a limited impact because of the biofilm intrinsic tolerance to drugs. Metabolism remodeling likely plays a central role during biofilm formation. Using comparative transcriptomics of different strains of Staphylococcus aureus and Enterococcus faecalis, we determined that almost all cellular adaptations are not shared between strains and species. Interestingly, we observed that the *de novo* purine biosynthesis pathway was upregulated during biofilm formation by both species in a specific medium. The requirement for purine could constitute an interesting new anti-biofilm target with a wide spectrum that could also prevent resistance evolution. These results are also relevant to a better understanding of the physiology of biofilm formation.

## INTRODUCTION

Biofilms are ubiquitous in the biosphere since approximately 80% of all bacteria adopt this lifestyle (1). These structures are formed by multicellular communities that adhere to a surface or an interface and are embedded in a matrix of extracellular polymeric substances (2, 3). The intrinsic properties of the matrixes confer them the ability to adapt to their environment and resist hostile conditions (2). Biofilms are known to cause significant problems in different aspects of human activity such as the food industry and the industrial and clinical sectors (4–7).

Biofilms are involved in a high number of nosocomial infections, approximately two-third of which are associated with some type of implanted medical devices (8). In hospitals, patients with biomedical implants such as prostheses are particularly at risk of developing severe complications related to biofilm-mediated infection during or after their surgery. These complications increase the morbidity and mortality rates of patients, as well as the duration and costs of hospitalization (9, 10). The most common pathogens isolated from those types of infection are Staphylococcus aureus, followed by *Enterococcus* spp., in approximately 36% and 19% of cases respectively (11, 12). Intrinsic properties of their biofilms provide 10 to 1,000 times more tolerance to antibiotics than the MIC required to kill their planktonic counterparts (10, 13–16). Therefore, biomedical catheter and implant infections caused by biofilm can be particularly difficult to treat because they are often unresponsive to antibiotic lock therapy, the currently recommended clinical practice to prevent and treat catheter-related infections (17).

S. aureus is a Gram-positive bacterium that naturally colonizes up to 60% of humans, but it can also become an opportunistic pathogen, causing a wide range of infections acquired in hospital or community environment (18). S. aureus biofilms often contain a matrix mainly composed of the polysaccharide intercellular adhesin (PIA), an important component of cell-cell adhesion (19). PIA production is regulated by the *icaADBC* operon (20) and is induced by high concentration of glucose and salt in the medium (21, 22). However, although most S. aureus strains possess the *ica* operon, some of them produce a PIA-independent biofilm (22). In these biofilms, the matrix is mostly composed of eDNA, MSCRAMMs (microbial surface components recognizing adhesive matrix molecules) and other large proteins that form fibrils for intercellular adhesion (23). Methicillin-resistant S. aureus and methicillin-sensitive S. aureus are more likely to produce PIA-independent and PIA-dependent biofilms, respectively (24, 25). Although S. aureus biofilms are well studied, some properties of PIA-dependent and PIA-independent biofilms are still poorly understood (26).

Enterococcus faecalis is a Gram-positive bacterium primarily found as a commensal organism that colonizes in low abundance in our gastrointestinal tract (27). In hospitals, E. faecalis can become an opportunistic pathogen causing several potentially lethal infections in susceptible patients (28). E. faecalis possesses a high level of tolerance toward environmental stress and antibiotic treatments (29–32), probably due to its ability to form a biofilm (33, 34). E. faecalis biofilm matrix is mostly composed of eDNA, polysaccharides, lipoteichoic acid, and extracellular proteases (35), but the underlying mechanisms leading to its formation remain to be clearly established.

Recent studies revealed a vast remodeling of metabolic pathways during the early phase of biofilm formation in Bacillus subtilis and Bacillus cereus, including modulation in fermentation processes, energy production, primary and secondary metabolism pathways (e.g., fatty acid, carbon, amino acid, and nucleotides metabolism) (36, 37). It is only recently that these pathways have been found to be involved in biofilm formation. In S. aureus, it has been reported that metabolic pathways such as the TCA cycle (38) and respiration (39) are positively or negatively regulated during biofilm formation. Diverse genes involved in metabolism were also transcriptionally modulated during biofilm formation in E. faecalis (40, 41). Identifying and understanding the cellular adaptation that bacteria need to perform when forming a biofilm could lead to the identification of new therapeutic targets to inhibit this process. Such targets would be of great help in designing therapeutic strategies to treat various chronic infections, to improve the effectiveness of current drugs and limit the development of tolerance.

Here, we used a transcriptomic approach to determine if specific cellular pathways were similarly regulated during biofilm formation in S. aureus and E. faecalis. Analysis of differently regulated genes in two strains of each species, and in two different media, allowed us to determine which metabolic pathways were consistently up- or downregulated during biofilm formation and whether the transcriptional regulation was conserved. Only the *de novo* purine biosynthesis pathway was commonly upregulated in all strains in one medium, and we validated the importance of this finding in S. aureus using gene disruption and drug treatment approaches.

## RESULTS

### Determination of the growth conditions for biofilm harvest.

Biofilms formed by various strains of S. aureus and E. faecalis can differ in their compositions in a natural environment or *in vitro* (35, 42). Consequently, to ensure that our assay includes commonly regulated cellular pathways in a strain-independent manner, we analyzed two strains of each pathogen. For S. aureus, we used the Methicillin-resistant *S. aureus* strain USA300 and the methicillin-sensitive *S. aureus* strain SH1000, which are phylogenetically apart (43). For E. faecalis, we selected two strains with robust biofilm-forming capacity; the vancomycin-resistant V583, which possess a plethora of mobile elements including three plasmids (44), and the widely referenced vancomycin-sensitive strain ATCC 29212 (45). A core genome analysis of these two E. faecalis strains using PATRIC Proteome Comparison Service (46) revealed that they share an average of 95.4% of protein sequence identity between their coding DNA sequence and 1082 conserved genes with 100% of sequence identity. Since only 36.3% of ATCC 29212 genes were perfectly conserved, it appears that both E. faecalis strains differ significantly, making them good candidates to identify genes whose regulation is well conserved. S. aureus strains share an average of 98.8% sequence identity and 2250 conserved genes with 100% of sequence identity (see Fig. S1A and Table S5 in the supplemental material). Interspecies comparisons showed that the S. aureus and E. faecalis strains have an average of 43.3% of sequence identity between their coding DNA sequence (see Fig. S1A and Table S5). The 455 genes with at least 50% homology between the four strains used in this study are involved in different metabolic pathways, such as amino acid, carbon, fatty acid or nucleotide metabolism (Fig. S1B). Conservation of these pathways between the two species is not surprising, considering that many metabolic subsystems are often conserved in prokaryotes (47).

**FIG 1:**
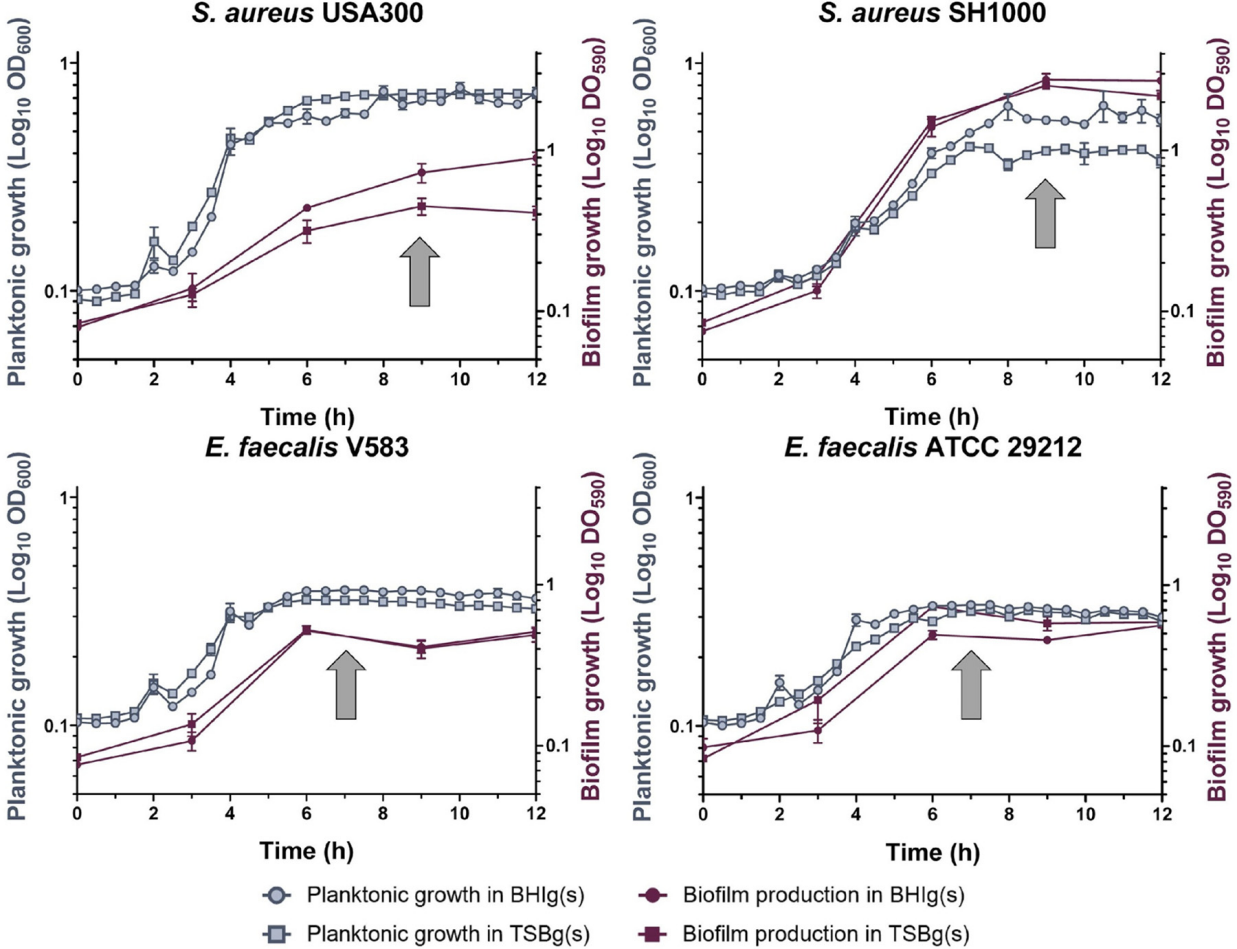
Growth dynamic of S. aureus and E. faecalis strains in a planktonic state or during biofilm formation. Planktonic growth (in blue) was performed at 37°C in shaking condition and measured by OD_600_, while biofilm growth (in red) was performed at 37°C without shaking and measured by crystal violet assay (OD_590_). Growth was observed in either BHIg(s) (circle) or TSBg(s) (square). Each point represents the mean of at least three biological replicates ± standard deviation (SD). RNA harvesting time is indicated by a gray arrow.

We analyzed mRNA expression levels during the early stage of biofilm development, which coincides with the most pronounced biofilm cellular adaptations in other Gram-positive species (36, 37). Targeting genes involved in these early stages of cellular adaptation might lead to a more efficient disruption of biofilm formation than targeting genes involved in later stages because matrix production would have already begun in the latter case. However, because early biofilm might be reached at different times by the different strains, we monitored biofilm formation using crystal violet to determine the best time point to harvest the mRNAs. As biofilms of S. aureus and E. faecalis reached their maximum development after 9 h and 7 h, respectively (Fig. 1), cells from both strains were harvested at the beginning of this plateau. In parallel, we monitored planktonic cell growth (OD_600_) under the same conditions but with agitation to harvest the cells just as they reached their growth plateau. These procedures demonstrated that in both conditions, growth dynamics were similar, and it ensured that the comparison of planktonic and biofilm cells were done at similar growth stages. Of note, we used two different media, tryptone soy broth (TSB) and brain heart infusion (BHI) to potentially influence biofilm matrix composition (48–50). Both media were supplemented with 0.5% glucose (TSBg and BHIg) to grow S. aureus USA300 and E. faecalis biofilms, and with 0.5% glucose and 3% sodium chloride to grow S. aureus SH1000 (TSBgs and BHIgs). Growth dynamic in BHIg(s) and TSBg(s) were comparable, except for S. aureus USA300, which formed a more abundant biofilm in BHIg.

### Transcriptomic analysis reveals global changes in gene expression during biofilm formation.

**FIG 2:**
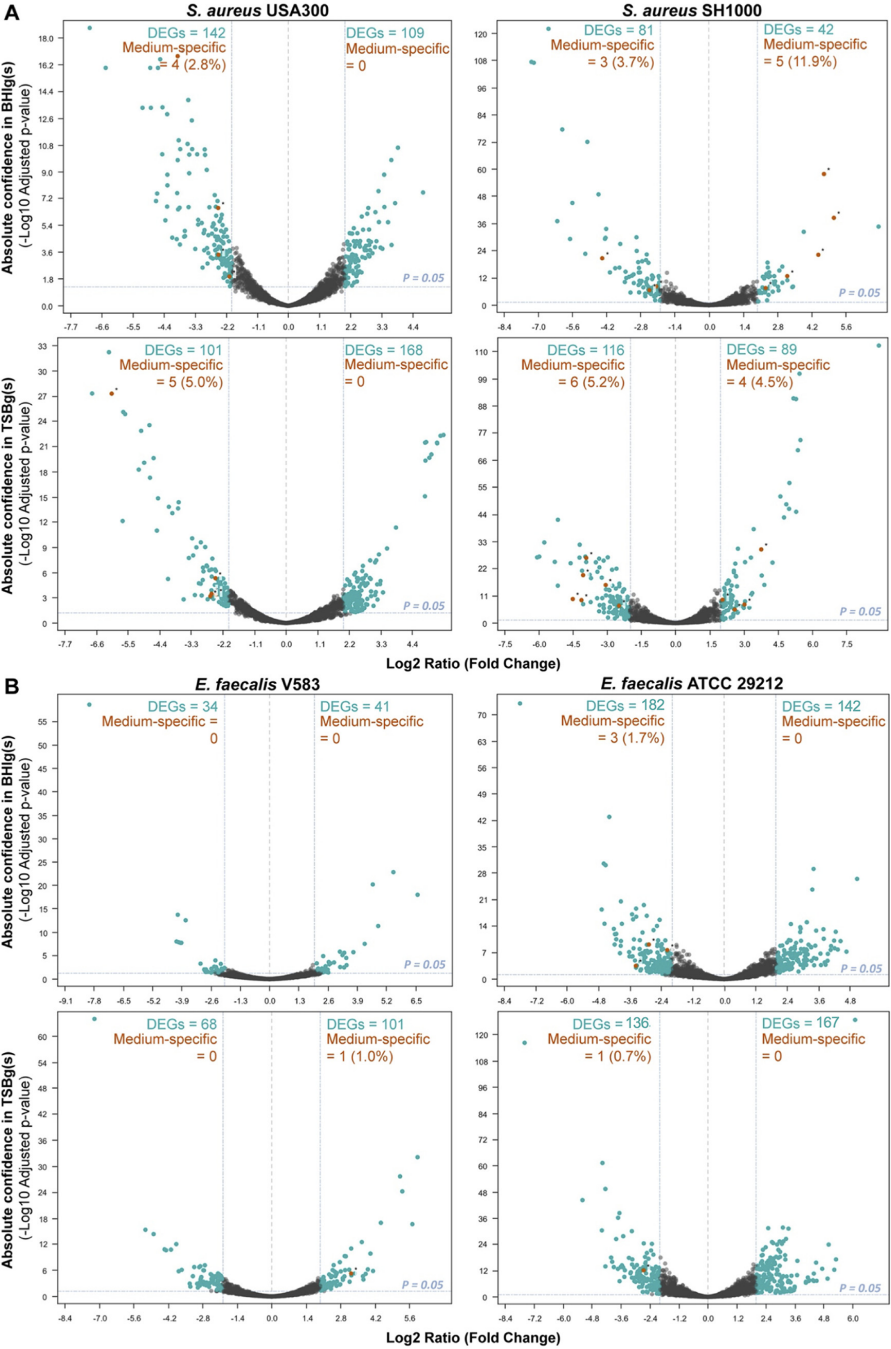
Transcriptional profiles of biofilm-grown cells and planktonic cells. The DEGs (blue dots) adjusted *P* value cutoff was 0.05. Genes were considered upregulated in biofilm if the log_2_ ratio (fold change) was ≥ 2.0 and downregulated in biofilm if the fold change was ≤ −2.0. DEGs from the media effect transcriptome analysis (BHIg[s] vs TSBg[s]) were then compared with the DEGs from the biofilm transcriptome analysis. Genes found in both analyzes are represented by orange dots and represent DEGs possibly emerging due to an effect of medium instead of biofilm formation. (A) S. aureus USA300 and S. aureus SH1000 DEGs in TSBg(s) or BHIg(s). (B) E. faecalis V583 and E. faecalis 29212 DEGs in TSBg or BHIg.

To determine changes in gene expression levels, we used an RNA-seq approach on cells harvested from planktonic and biofilm conditions from biological triplicates of each experimental condition. A principal-component analysis of differential expression showed that biological replicates mostly clustered together (see Fig. S2 in the supplemental material). Genes were considered differentially expressed in biofilm versus planktonic when showing a statistical significance (adjusted *P* value < 0.05) and a fold change (log_2_ ratio) ≥ |2.0|. All media combined, 466 unique differentially expressed genes (DEGs; ∼17.3% of the genome) were found for S. aureus USA300, 280 unique DEGs (∼9.6% of the genome) for S. aureus SH1000, 242 unique DEGs (∼7.7% of the genome) for E. faecalis V583 and 461 unique DEGs (∼16.4% of the genome) for E. faecalis ATCC 29212 (see Table S4 and Gene Expression Omnibus database under the accession number GSE162709). Upregulated (log_2_ fold change ≥ 2.0) and downregulated (log_2_ fold change ≤ −2.0) DEGs distribution among S. aureus and E. faecalis strains and media are displayed in the Volcano plots shown in Fig. 2.

### Media effect on gene expression.

Metabolic pathways have been previously shown to be involved in biofilm formation in several Gram-positive bacteria (36, 37, 51), but these pathways can also be modulated by nutrients in the growth medium. Thus, we examined the influence of two different biofilm growth media on gene expression patterns to determine if the regulation we observed was specific to the medium or to biofilm formation. To do that, we determined genes that were differentially expressed in TSBg(s) compared to BHIg(s), all growth condition combined. For S. aureus, 75 genes of USA300 and 149 genes of SH1000 had their expression significantly modulated by the two media. Of these, a total of 9 and 18 genes were also found differentially expressed in biofilms for USA300 and SH1000, respectively (Fig. 2). For E. faecalis strains, we observed that only 11 genes of V583 and 31 genes of ATCC 29212 were differentially expressed in one or the other media, all growth conditions combined. Consequently, the growth medium used had a small effect on the gene expression pattern of E. faecalis strains during biofilm formation, with less than 1% of all DEGs being also differentially expressed when comparing the transcriptomes obtained from different growth media. Altogether, our results suggest that the medium has a minor influence on gene expression during biofilm formation, but this difference needs to be considered.

### Gene set enrichment analysis reveals global cellular adaptations during biofilm formation.

**FIG 3:**
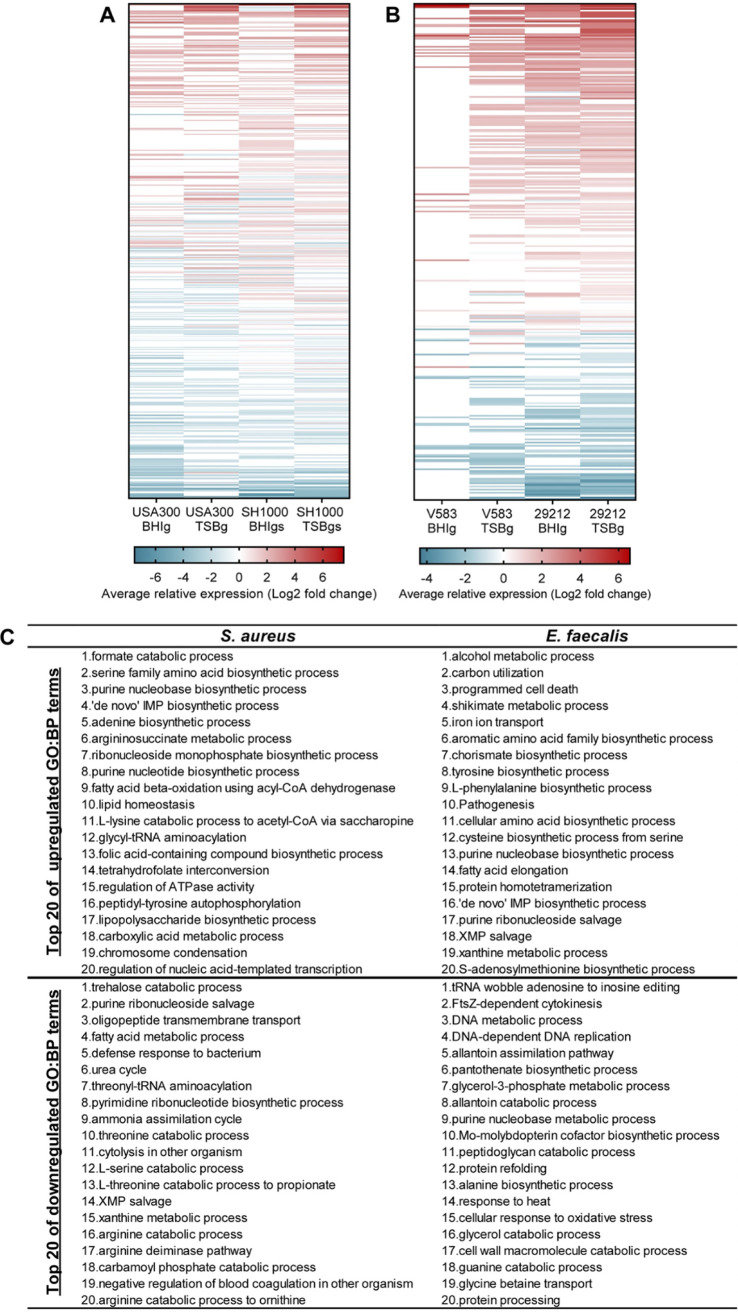
Average relative expression of GO:BP terms during biofilm formation. Each row of both heatmaps represents a single functional annotation term from DAVID Bioinformatic software using GO:BP annotation source. A term’s relative expression is the average of the log_2_ fold change of every associated gene with an adjusted *P* value cutoff was 0.05. Genes were considered upregulated in biofilm if the log_2_ ratio (fold change) was ≥ 2.0 and downregulated in biofilm if the fold change was ≤ −2.0. DEGs from the media effect transcriptome analysis (BHIg[s] vs TSBg[s]) were then compared to the DEGs from the biofilm transcriptome analysis. Genes found in both analyzes are represented by orange dots and represent DEGs possibly emerging due to an effect of medium instead of biofilm formation. (A) S. aureus USA300 and SH1000, in BHIg(s) or TSBg(s) medium. (B) Functional annotation terms’ (total = 316) relative expression in E. faecalis V583 and 29212, in BHIg or TSBg medium. (C) Table of the top 20 upregulated and downregulated GO:BP terms for each species, by calculating the average of the relative expression of each term for the 2 strains in each medium.

We compared biofilm and planktonic gene expression patterns from the four strains in TSBg(s) or BHIg(s) using DAVID Bioinformatic resources (52). Since S. aureus USA300 and E. faecalis ATCC 29212 locus tags were not recognized by DAVID, we attributed the corresponding recognized locus tag of their species counterpart based on sequence homology and orthologs with the help of BioCyc Pathway tools version 23.0 (53) and AureoWiki (54). Genes up- or downregulated in the heatmap of S. aureus and E. faecalis represented 497 and 316 Gene Ontology:biological process (GO:BP) terms, respectively (Fig. 3A and B). The 20 most up- and downregulated GO:BP terms for each species, all strains and media combined, are shown in Panel C of Fig. 3 as a portrait of species-specific global regulation. The same analysis was performed with the Kyoto Encyclopedia of Genes and Genomes (KEGG) pathways as the annotation source showed that 103 KEGG functions of S. aureus and 97 KEGG functions of E. faecalis were represented in up- or downregulated genes (Fig. S3 in the supplemental material). Many metabolism-related pathways were upregulated during biofilm formation, all annotation sources combined, such as carbon, fatty acid, nucleotides and some amino-acid metabolism genes. However, many of these pathways appeared differently regulated according to the growth medium, as shown in Fig. S4, which depicts the most up- and downregulated GO:BP terms by species and by medium.

### Absence of common cellular adaptation between S. aureus and E. faecalis strains during biofilm formation.

The differences in GO:BP terms up- and downregulated in S. aureus and E. faecalis suggested that genes differently regulated during biofilm formation are very different between both species. To confirm this observation, we compiled all the genes showing a statistically significative (adjusted *P* value < 0.05) fold change of (log_2_ ratio) ≥ |1.0| in both media and for both strains of each species (Table S3 in the supplemental material). For S. aureus, 26 genes were consistently up- or downregulated in all conditions tested. Interestingly, genes encoding for urease accessory proteins were significantly upregulated in USA300 and downregulated in SH1000. Previous studies have reported that urease genes were upregulated in mature biofilms formed by UAMS-1 and SA113 (20, 55). We also observed strong upregulation of the formate dehydrogenase gene *fdh* and of the DNA-binding protein HU gene *hup* in both media, similarly to what was observed previously (55). Our observations correlate with a recent study showing that S. aureus gene expression within the biofilm is largely strain-dependent (56). For E. faecalis a total of 50 genes, including many transporters, were either up- or downregulated. Several of these genes were also previously reported as being modulated in biofilm, such as the downregulation of *nusA* and of the cation-transporting ATPase EF0871 (40), and the upregulation of the S-adenosylmethionine synthetase gene *metK* and of the quorum sensing gene *luxS* (57). Importantly, exhaustive examination of the significantly regulated genes in biofilms in all conditions revealed no pathways for which gene regulation was common to both species.

### Purine biosynthesis remodeling during biofilm formation.

**FIG 4:**
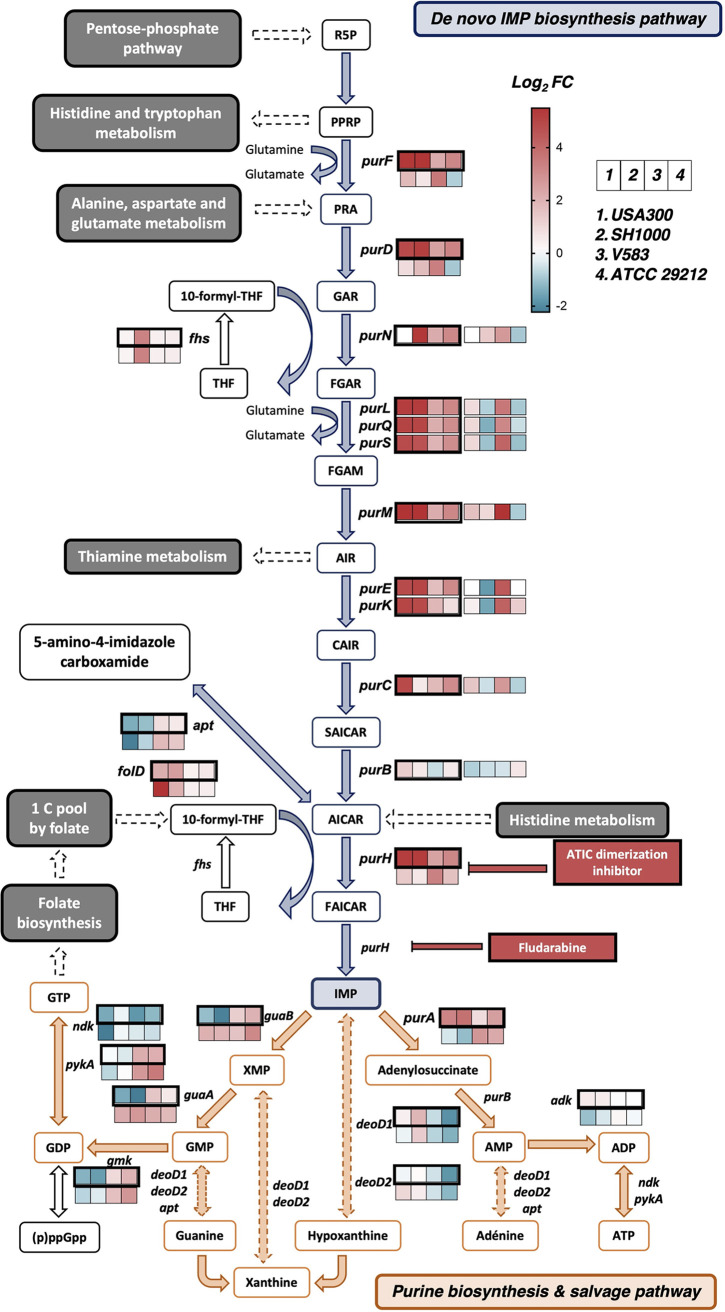
The *de novo* purine biosynthesis pathway is strongly upregulated during biofilm formation in TSBg(s). The purine biosynthesis pathway includes the *de novo* purine biosynthesis pathway (in blue) and the purine salvage pathway (in orange). Plain arrows indicate a unique reaction while dotted arrows indicate multiple reactions. The expression level (log_2_ fold change) during biofilm formation of the genes involved in the pathway, as obtained by the transcriptomic analysis, are indicated by a 4-square stripe (from left to right: S. aureus USA300, S. aureus SH1000, E. faecalis V583 and E. faecalis 29212). The stripe with thick black lines represents expression in TSBg(s), and the thin line is in BHIg(s). The red and blue color scale indicates high and low expression levels, respectively. For abbreviations, see Table S6.

Bacteria can metabolize purines *de novo* from 5′-phosphoribosylamine-1-pyrophosphate through biosynthesis of IMP (IMP), or they can recycle purines from the nucleic acids present in their environment via the purine salvage pathway (58). Interestingly, GO:BP terms related to purine biosynthesis were among the 20 most upregulated term between both species when the two media were examined together (Fig. 3B). This metabolic pathway, with each gene involved in these reactions, is depicted in Fig. 4. In adjacent boxes we show their expression level in biofilm (log_2_ fold change) in TSBg(s) (dark outline) and in BHIg(s) (thin outline). Overall, genes in the *de novo* purine biosynthesis pathway were homogeneously strongly upregulated (log_2_ fold change ≥ 2.0) in all S. aureus and E. faecalis strains in TSBg(s), and also in BHIg for E. faecalis V583. Only *purB*, found also in the purine salvage pathway, did not show a strong upregulation. Interestingly, we did not find significant and conserved regulation of the purine biosynthesis regulator PurR, suggesting that another mechanism is involved here. Gene expression levels of the purine salvage pathway were heterogeneously and weakly regulated.

### Genes involved in the purine biosynthesis pathway are important for biofilm production.

**FIG 5:**
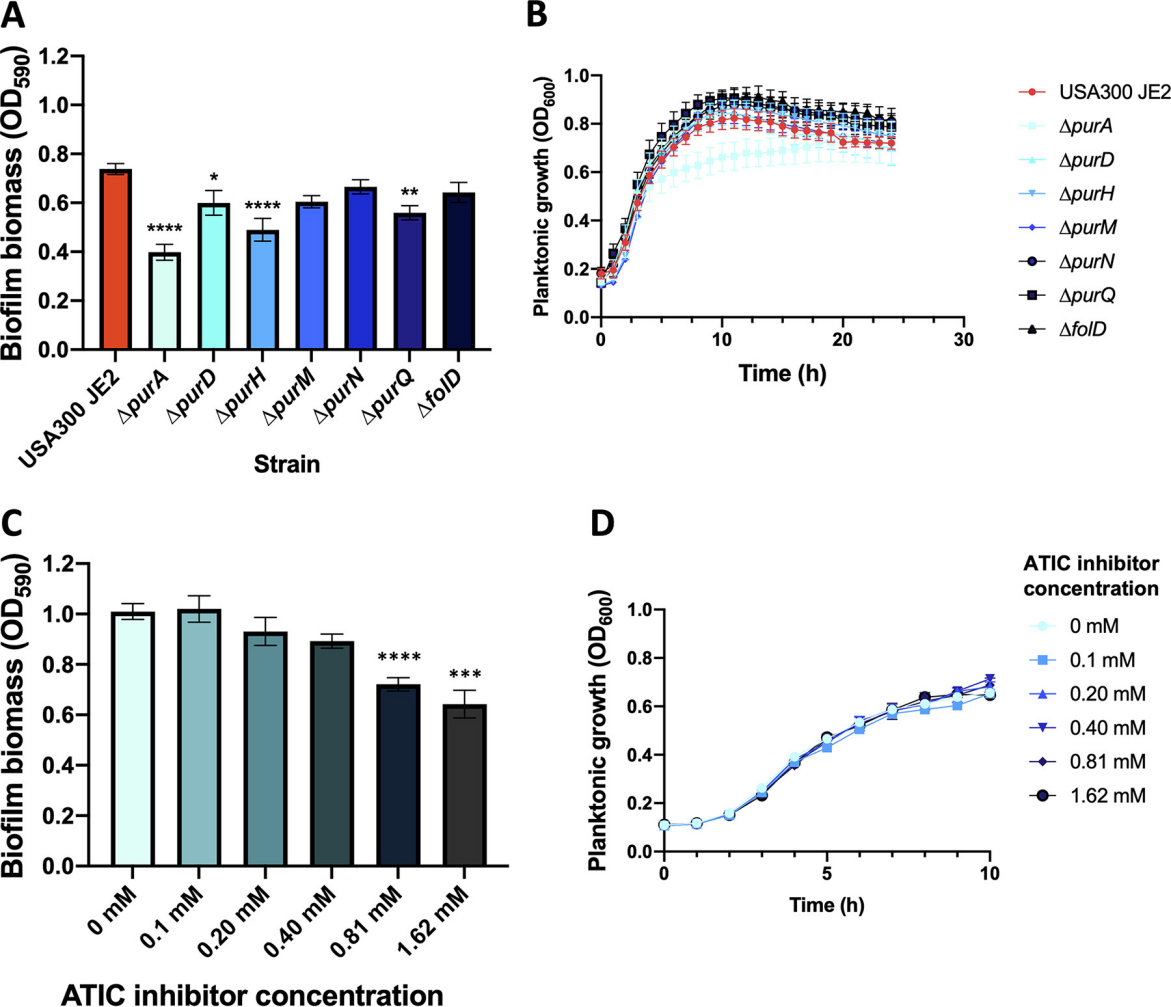
Inhibition of *de novo* purine biosynthesis pathway affects biofilm formation in S. aureus. (A) Biofilms of S. aureus USA300 containing transposon insertion (NTML) in gene encoding for the purine biosynthesis pathway were formed in TSBg and quantified by crystal violet. Each bar represents biofilm production (in OD_590_) of 9 biological replicates ± SEM (one-way ANOVA and Dunnett’s multiple comparison; *, *P* value < 0.05; **, *P* value < 0.01; ****, *P* value < 0.0001). (B) Planktonic growth of S. aureus USA300 mutants in TSBg. Each point of the curve represents the cell density (OD_600_) of 9 biological replicates ± SEM. (C) Biofilms of S. aureus SH1000 were formed in TSBgs containing different concentration of ATIC dimerization inhibitor and quantified by crystal violet. Each bar represents biofilm production (in OD_590_) of at least 3 biological replicates ± SD (one-way ANOVA and Dunnett’s multiple comparison; ***, *P* value < 0.001; ****, *P* value < 0.0001). (D) Planktonic growth of S. aureus SH1000 in the presence of various concentration of ATIC inhibitor in TSBgs. Each point of the curve represents the cell density (OD_600_) of at least 3 biological replicates ± SD.

Since *de novo* purine biosynthesis was found highly upregulated in biofilm formation in TSBg(s) for all the strains used here, we wanted to confirm that this pathway is important for S. aureus biofilm formation. Thus, we performed biofilm growth assays using S. aureus USA300 strains with transposon insertion in *purD*, *purH*, *purM*, *purN*, and *purQ* genes from the purine biosynthesis pathway (Nebraska Transposon Mutant Library; NTML) (59). We also used a strain with a transposon insertion in *purA* from the purine biosynthesis and salvage pathway and in *folD* from the 1 C pool by folate pathway. The bifunctional protein FolD synthetizes 10-formyl tetrahydrofolate (10-formyl-THF), a cofactor used by *purN* and *purH* (60). As shown in Fig. 5A, disruption of *purH* and *purQ* significantly impaired biofilm production (Fig. 5A), and this effect was not due to a delayed or decreased cell division rate (Fig. 5B). This defect in biofilm formation was also visible under confocal microscopy (Fig. S6); *ΔpurH* had a generally weaker biofilm, and *ΔpurQ* biofilm showed good exopolysaccharide signal but appeared to contain less cells. Interruption of *purA*, a gene also part of the salvage pathway, had a strong impact on biofilm formation (Fig. 5A). Its biofilm appeared very sparse and contained few cells (Fig. S6). However, this mutation also had a mild effect on planktonic growth (Fig. 5B), which could partly explain the effect on biofilm production. Importantly, complementation *of ΔpurA*, *ΔpurH*, and *ΔpurQ* by addition of the final product of the pathway IMP restored biofilm formation to wild-type levels (Fig. S5 in the supplemental material). USA300 biofilm formation relies heavily on eDNA, thus possibly exacerbating the necessity of a functional purine biosynthesis pathway. To assess the importance of this pathway in PIA-dependent biofilms, we treated SH1000 with ATIC dimerization inhibitor, a drug targeting the bifunctional enzyme PurH (61). As shown in Fig. 5C, this drug successfully reduced biofilm formation by SH1000 without affecting cell growth (Fig. 5D). While the high concentration of drugs required is likely because the ATIC inhibitor was developed specifically for cancer treatment in mammalian cells, this result supports the notion that the purine biosynthesis pathway constitutes an interesting target to reduce biofilm formation in S. aureus.

## DISCUSSION

Recent developments in biofilm research have highlighted metabolic remodeling during biofilm formation in some Gram-positive bacteria (36, 37). However, regulatory mechanisms leading to biofilm formation are often species or even strain-specific, making it difficult to develop anti-biofilm strategies that target a wide spectrum of bacteria (62, 63). Our transcriptomic approach with Gram-positive strains of clinical importance confirm that no cellular pathways or genes are commonly regulated in the two strains of S. aureus and E. faecalis during biofilm formation in both media tested (Table S3 in the supplemental material).

TSB and BHI medium supplemented with glucose and/or sodium chloride are two frequently used media to grow S. aureus and E. faecalis biofilms (49, 64). However, it was suggested that BHI promotes a protein-based matrix, while TSB-based media promotes either a PIA-dependent or PIA-independent biofilm (48). According to our transcriptomic analysis, several genes were found differentially expressed in one or the other medium, all strains and growth conditions combined. Many of those DEGs were attributed to primary metabolism pathways and some to secondary metabolism pathways (Table S4 in the supplemental material). We observed that the growth media used for biofilm formation had some effect on the transcriptome profiles in S. aureus, possibly due to environmental factors, but that these profiles were mostly unchanged in E. faecalis strains (Fig. 2). However, a recent study in E. faecalis highlights that the genetic determinants for robust biofilm formation in the OG1RF strain differ according to the medium and the experimental conditions (65). Different growth media were also reported to influence biofilm phenotypic properties and matrix composition in other Gram-positive strains such as B. subtilis (66).

Some of the genes and biological functions we found upregulated in S. aureus during biofilm growth (Fig. 3), such as fermentation, nucleotide metabolism and the TCA cycle, were already observed in other transcriptomic, metabolomic or proteomic studies using other S. aureus strains (55, 67–70). In E. faecalis, only a few metabolic adaptations during biofilm formation were reported (40, 57). Therefore, our data provide new information about biofilm formation of E. faecalis that could be exploited in further studies and consolidate previous observations on S. aureus biofilm formation.

Several studies previously reported purine biosynthesis as being upregulated in both Gram-positive and Gram-negative bacteria during biofilm formation (36, 51, 71–75). In keeping with those studies, we found a strong upregulation of the *de novo* purine biosynthetic process during biofilm formation in E. faecalis and S. aureus strains in TSBg(s) (Fig. 4). Inhibition of this pathway either by genetic disruption or by using a drug targeting a key enzyme led to a decrease in S. aureus USA300 and SH1000 biofilm production (Fig. 5). These results points toward an important need for purine during biofilm formation, which would be reflected by an upregulation of *de novo* purine biosynthesis in an environment where this nutrient is not abundant. An interesting perspective from this study will be to use drugs targeting this pathway to investigate this phenomenon in other Gram-positive strains that lack efficient transformation tools or an available mutant library.

Purines are required for eDNA production, an important constituent of the extracellular matrix of S. aureus (76), E. faecalis (77) and other Gram-positive bacteria such as B. subtilis or B. cereus (78). However, it was reported that in B. cereus, *purD* and *purH* deletions did not significantly affect the level of eDNA in B. cereus matrix compared to the wild-type strain (51). Importantly, genes of the *de novo* purine biosynthesis pathway were shown to be important for biofilm formation in Streptococcus sanguinis (75), Pseudomonas fluorescens (74), and a *Burkholderia* symbiont (72). Recent studies also showed that upregulation of the *de novo* purine biosynthesis pathway led to increased virulence in mice (79–81). Our results show that this pathway is important in S. aureus and is upregulated in E. faecalis and indicate that the requirement for high amounts of purine during biofilm formation might be widespread among bacteria.

## MATERIALS AND METHODS

### Strains and media.

Strains used for the transcriptomic analysis are S. aureus USA300, S. aureus SH1000, E. faecalis V583, and E. faecalis ATCC 29212 (a kind gift from F. Malouin, Université de Sherbrooke). Biofilm phenotypic assays were performed with S. aureus USA300 mutants from the Nebraska Transposon Mutant Library. The USA300 JE2 strain was used as a wild-type control (NTML; a kind gift from J.-P. Côté, Université de Sherbrooke) (82) (see Table S1 in the supplemental material). Strains were maintained on Tryptic Soy agar or Brain Heart Infusion agar plates, and for the NTML mutants, 5 µg/mL of erythromycin was added to the media. To induce biofilm formation, Tryptic Soy broth or BHI broth supplemented with 0.5% (wt/vol) glucose (TSBg or BHIg) was used for E. faecalis strains and S. aureus USA300, and TSBg or BHIg supplemented with 3.0% (wt/vol) sodium chloride (TSBgs or BHIgs) was used for S. aureus SH1000.

### Growth measurement (planktonic cells and biofilm).

Colonies from overnight cultures on Tryptic Soy agar or BHI agar plates at 37°C were suspended in 1 mL of the appropriate biofilm inducing medium. Then, 200 µL of biofilm inducing medium was added in wells of a 48-wells culture plate and inoculated to an OD_600_ of 0.005 with the bacterial suspension. For planktonic growth measurement (at least three biological replicates), plates were incubated in a Synergy HT plate reader (BioTek, Winooski, VT, USA) at 37°C with continuous agitation at 1,140 rpm to prevent biofilm formation and monitored at 600 nm every 30 min for 12 h (Fig. 1), 24 h (Fig. 5B), or 10 h (Fig. 5D). Absence of biofilm in these conditions was confirmed by crystal violet. For Fig. 5C and D, ATIC dimerization inhibitor (Millipore-Sigma) was added from a 1 mg/mL stock in distilled water.

For biofilm formation, polystyrene plates were incubated at 37°C without agitation for 12 h (Fig. 1), 24 h (Fig. 5A) or 9 h (Fig. 5C); quantification (at least 3 biological replicates) was performed using the crystal violet method (83). Briefly, biofilms were gently washed with phosphate-buffered saline (PBS), covered with 200 µL of 0.01% (wt/vol) crystal violet and incubated for 20 min at room temperature, protected from light. Each well was then gently washed with sterile distilled water to remove the excess of dye. Stained biofilms were solubilized in 200 µL of 33% acetic acid and quantified by spectrophotometry at 590 nm.

### mRNA extraction and isolation.

Planktonic cells (three biological replicates) were washed with cold PBS and kept in RNA stabilization solution (25 mM sodium citrate, 10 mM EDTA, 47% ammonium sulfate, adjusted to pH 5.2) until all samples were harvested. Biofilms (three biological replicates) were washed with cold PBS and then suspended in RNA stabilization solution by scraping the bottom of the well. Planktonic and biofilms cells were then centrifuged, the supernatant discarded, and the pellet kept at −80°C until RNA extraction.

Cells were suspended in 250 µL of fresh lysis buffer (45 mg/mL of lysozyme, 16 µL/mL of lysostaphin 5 mg/mL, and 10 µL/mL of mutanolysin 5 U/µL in Tris-EDTA) and incubated for 1 h at 37°C, while mixed by inversion every 15 min. They were then transferred in O-Ring tubes with 25–50 mg of acid-washed beads and mixed with three volumes of TRI Reagent, to be lysed mechanically with a FastPrep-24 Classic (MP Biomedicals, Santa Anna, CA, USA) (speed of 4.5 m/s, 2 × 20 s). Total RNA was extracted with the Direct-zol RNA Miniprep Plus kit (Zymo Research, Irvine, CA, USA) following the manufacturer's instruction, including the DNase I (New England Biolabs, Ipswich, MA, USA) treatment in the extraction column. RNA quantity and the integrity number were evaluated with an Agilent 2100 Bioanalyzer (Agilent Technologies, Santa Clara, CA, USA), and all samples had integrity number between 8 and 10. To remove rRNA (rRNA), samples were treated using a Pan-Prokaryote riboPOOL kit (siTOOLs Biotech Gmbh, Planegg, Germany) coupled with streptavidin magnetic beads (New England Biolabs, Ipswich, MA, USA). mRNA was eluted in 50 µL of nuclease-free water and then cleaned and concentrated using a Clean & Concentrator kit (Zymo Research, Irvine, CA, USA). mRNA samples were kept at −20°C if used on the same day or frozen at −80°C for long-term storage. Ribo-depletion success was assessed by verifying the diminution or disappearance of the 16s rRNA and 23s rRNA peaks with an Agilent 2100 Bioanalyzer, between a treated sample and a nontreated sample.

### Construction of prokaryotic Illumina libraries.

Libraries were prepared for RNA sequencing according to the instructions of the NEBNext Ultra II Directional RNA Library Prep Kit for Illumina (New Englands Biolabs, Ipswich, MA, USA), with the following exception. RNA samples were fragmented 7 min instead of 15 min to produce fragments longer than 100 bp. Instead of the adaptors provided with the kit, we used TruSeq compatible YIGA adaptors (see Table S2 in the supplemental material). Nucleic acid purification was performed using Mag-Bind TotalPure NGS beads (Omega Bio-Tek Inc, Norcross, GA, USA) following the recommended ratio in the NEBNext kit. Ligation products were subjected to PCR amplification using a reverse primer containing a unique 8 bp index for each sample (see Table S2 in the supplementary material). The concentration of nucleic acid in the libraries was measured using a Quant-iT PicoGreen dsDNA assay kit (Invitrogen, Carlsbad, CA, USA) and 1.13 ng of each sample were pooled in 80 µL of nuclease-free water. Quantity and quality of the final mix were confirmed with an Agilent 2100 Bioanalyzer before submitting to a Nextseq500 High Output 75 bp sequencing.

### RNA sequencing data analysis.

Approximately 8,000,000 reads per library were obtained using an Illumina Nextseq system providing single-ended reads of 75 bp each (Rnomics platform, Université de Sherbrooke). The data was analyzed using Geneious Prime 2020.0.3 (https://www.geneious.com). Fastq files were trimmed with the BBDuk plugin using the default parameters and eliminating reads shorter than 10 bp. Trimmed data were then aligned with Bowtie2 (84) using an “end-to-end” alignment type, a “High Sensitivity/Medium” preset and using the “do not trim option,” against the following reference chromosomes: GenBank accession number GCA_000013425.1 (for S. aureus SH1000, a close descendant of the ancestral strain NCTC 8325) (85); GenBank accession number GCA_000017085.1 (for S. aureus USA300); GenBank accession number GCA_000742975.1 (for E. faecalis ATCC 29212) and GenBank accession number GCA_000007785.1 (for E. faecalis V583). Finally, the differentially expressed genes (DEGs) were assessed with DESeq2 (86) by combining the 3 biological replicates of each condition and comparing biofilm cultures vs planktonic cultures in either TSBg(s), BHIg(s) or both. Only the genes with an adjusted *P* value ≤ 0.05 were retained for further analysis. DEGs were scored as upregulated if they had a log_2_ fold change ≥ 2.0 and downregulated if they had a log_2_ fold change ≤ −2.0. For the transcriptomic study on growth media effect on genes expression, DEGs with a log_2_ fold change ≥ 2.0 were considered upregulated in BHIg(s) while DEGs with a log_2_ fold change ≤ −2.0 were considered upregulated in TSBg(s).

### Functional annotation.

The various gene set enrichment analysis (GSEA) (or functional annotation) was performed using DAVID Bioinformatics Resources version 6.8 (52). The locus tag of the upregulated and downregulated DEGs were submitted as a list to DAVID and then analyzed with the Functional Annotation tool. The UP_KEYWORDS of the Functional_category, the KEGG_PATHWAY of the Pathways category, the GOTERM_BP_DIRECT and/or GOTERM_MF_DIRECT of the Gene_Ontology category were selected as possible annotation sources. The analysis was performed with the functional annotation chart, including the default thresholds of a count of 2 and an EASE of 0.1 and applying the following statistical test: fold enrichment, Benjamini, and FDR. Following DAVID recommendations, function with a fold enrichment ≥ 1.5 and a Benjamini value < 0.05 were considered interesting. The GSEA comparing biofilm vs planktonic conditions was confirmed with the ShinyGO v0.61 software. The locus tags of the same DEGs were submitted as a list, using either the E. faecalis STRINGdb or the S. aureus NCTC8325 STRINGdb and the default *P* value cutoff (FDR) of 0.05. Both KEGG pathways and GO:BP were considered.

### Core genome circular comparison.

To identify potential conserved cellular pathways among the strain used in this study, each of them was submitted to the Proteome Comparison Service performs, a protein sequence-based genome comparison tool from PATRIC (46), using the default parameters and the following reference genomes: Staphylococcus aureus subsp. *aureus* NCTC 8325 (Genome ID: 93061.5), Staphylococcus aureus subsp. *aureus* USA300_TCH1516 (Genome ID: 451516.9), Enterococcus faecalis V583 (Genome ID: 226185.9) and E. faecalis ATCC 29212 (Genome ID: 1201292.8). Genome comparison was illustrated in a scalable vector graphic image file produced by Circos. All the recognized locus tags of the genes sharing over 50% of sequence identity in the genome comparison text file of every strain were subjected to a GSEA as described earlier.

### Statistical analysis.

Statistical analysis was performed using GraphPad Prism 8.4.0. Comparisons were done using Student's *t* test or one-way ANOVA followed by Tukey’s multiple-comparison test, both with 95% confidence intervals.

### Data availability.

The data sets generated and analyzed during the current study are available in the Gene Expression Omnibus database under the accession number GSE162709.

## References

[1] Flemming H-C, Wuertz S. 2019. Bacteria and archaea on Earth and their abundance in biofilms. Nat Rev Microbiol 17:247–260. doi:10.1038/s41579-019-0158-9. 30760902

[2] Flemming H-C, Wingender J, Szewzyk U, Steinberg P, Rice SA, Kjelleberg S. 2016. Biofilms: an emergent form of bacterial life. Nat Rev Microbiol 14:563–575. doi:10.1038/nrmicro.2016.94. 27510863

[3] Donlan RM. 2002. Biofilms: Microbial life on surfaces. Emerg Infect Dis 8:881–890. doi:10.3201/eid0809.020063. 12194761 PMC2732559

[4] Galié S, García-Gutiérrez C, Miguélez EM, Villar CJ, Lombó F. 2018. Biofilms in the food industry: Health aspects and control methods. Front Microbiol 9:898. doi:10.3389/fmicb.2018.00898. 29867809 PMC5949339

[5] Sahoo D, Bhatt M, Jena S, Dash D, Chayani N. 2015. Study of biofilm in bacteria from water pipelines. J Clinical and Diagnostic Res 9:9–11. 10.7860/JCDR/2015/12415.5715 PMC4413065 25954617

[6] Høiby N, Ciofu O, Johansen HK, Song Z, Moser C, Jensen PØ, Molin S, Givskov M, Tolker‐Nielsen T, Bjarnsholt T. 2011. The clinical impact of bacterial biofilms. Int J Oral Sci 3:55–65. doi:10.4248/IJOS11026. 21485309 PMC3469878

[7] Costerton JW, Cheng KJ, Geesey GG, Ladd TI, Nickel JC, Dasgupta M, Marrie TJ. 1987. Bacterial biofilms in nature and disease. Annu Rev Microbiol 41:435–464. doi:10.1146/annurev.mi.41.100187.002251. 3318676

[8] Bryers JD. 2008. Medical biofilms. Biotechnol Bioeng 100:1–18. doi:10.1002/bit.21838. 18366134 PMC2706312

[9] van Epps JS, Younger JG. 2016. Implantable device-related infection. Shock 46:597–608. doi:10.1097/SHK.0000000000000692. 27454373 PMC5110396

[10] Lewis K. 2001. Riddle of biofilm resistance. Antimicrob Agents Chemother 45:999–1007. doi:10.1128/AAC.45.4.999-1007.2001. 11257008 PMC90417

[11] Arciola CR, Campoccia D, Montanaro L. 2018. Implant infections: adhesion, biofilm formation and immune evasion. Nat Rev Microbiol 16:397–409. doi:10.1038/s41579-018-0019-y. 29720707

[12] Public Health Agency of Canada. 2014. Central venous catheter-associated blood stream infections in intensive care units in Canadian acute-care hospitals: Surveillance report. Centre for Communicable Diseases and Infection Control, Public Health Agency of Canada.

[13] Høiby N, Bjarnsholt T, Givskov M, Molin S, Ciofu O. 2010. Antibiotic resistance of bacterial biofilms. Int J Antimicrob Agents 35:322–332. doi:10.1016/j.ijantimicag.2009.12.011. 20149602

[14] Khatoon Z, McTiernan CD, Suuronen EJ, Mah TF, Alarcon EI. 2018. Bacterial biofilm formation on implantable devices and approaches to its treatment and prevention. Heliyon 4:e01067. doi:10.1016/j.heliyon.2018.e01067. 30619958 PMC6312881

[15] Lebeaux D, Ghigo J-M, Beloin C. 2014. Biofilm-related infections: Bridging the gap between clinical management and fundamental aspects of recalcitrance toward antibiotics. Microbiol Mol Biol Rev 78:510–543. doi:10.1128/MMBR.00013-14. 25184564 PMC4187679

[16] Davies D. 2003. Understanding biofilm resistance to antibacterial agents. Nat Rev Drug Discov 2:114–122. doi:10.1038/nrd1008. 12563302

[17] Justo JA, Bookstaver PB. 2014. Antibiotic lock therapy: Review of technique and logistical challenges. Infect Drug Resist Dec 12:343–363. 10.2147/IDR.S51388 PMC4271721 25548523

[18] Kluytmans J, van Belkum A, Verbrugh H. 1997. Nasal carriage of *Staphylococcus aureus*: epidemiology, underlying mechanisms, and associated risks. Clin Microbiol Rev 10:505–520. doi:10.1128/CMR.10.3.505. 9227864 PMC172932

[19] Schilcher K, Horswill AR. 2020. Staphylococcal biofilm development: structure, regulation, and treatment strategies. Microbiol Mol Biol Rev 84:1–36. doi:10.1128/MMBR.00026-19. PMC7430342 32792334

[20] Beenken KE, Dunman PM, McAleese F, Macapagal D, Murphy E, Projan SJ, Blevins JS, Smeltzer MS. 2004. Global gene expression in *Staphylococcus aureus* Biofilms. J Bacteriol 186:4665–4684. doi:10.1128/JB.186.14.4665-4684.2004. 15231800 PMC438561

[21] Cue D, Lei MG, Lee CY. 2012. Genetic regulation of the intercellular adhesion locus in staphylococci. Front Cell Infect Microbiol 2:38. doi:10.3389/fcimb.2012.00038. 23061050 PMC3459252

[22] Fitzpatrick F, Humphreys H, O'Gara JP. 2005. Evidence for icaADBC-Independent biofilm development mechanism in methicillin-resistant *Staphylococcus aureus* clinical isolates. J Clin Microbiol 43:1973–1976. doi:10.1128/JCM.43.4.1973-1976.2005. 15815035 PMC1081404

[23] Otto M. 2013. Staphylococcal infections: Mechanisms of biofilm maturation and detachment as critical determinants of pathogenicity. Annu Rev Med 64:175–188. doi:10.1146/annurev-med-042711-140023. 22906361

[24] O’Gara JP. 2007. Ica and beyond: Biofilm mechanisms and regulation in *Staphylococcus epidermidis* and *Staphylococcus aureus*. FEMS Microbiology Lett 270:179–188. doi:10.1111/j.1574-6968.2007.00688.x. 17419768

[25] McCarthy H, Rudkin JK, Black NS, Gallagher L, O'Neill E, O'Gara JP. 2015. Methicillin resistance and the biofilm phenotype in *Staphylococcus aureus*. Front Cell Infect Microbiol 5:1–9. doi:10.3389/fcimb.2015.00001. 25674541 PMC4309206

[26] Mlynek KD, Bulock LL, Stone CJ, Curran LJ, Sadykov MR, Bayles KW, Brinsmade SR. 2020. Genetic and biochemical analysis of CodY-mediated cell aggregation in *Staphylococcus aureus* reveals an interaction between extracellular DNA and polysaccharide in the extracellular matrix. J Bacteriol 202:e00593-19. doi:10.1128/JB.00593-19. 32015143 PMC7099133

[27] Willett JLE, Ji MM, Dunny GM. 2019. Exploiting biofilm phenotypes for functional characterization of hypothetical genes in *Enterococcus faecalis*. NPJ Biofilms Microbiomes 5:1–14. doi:10.1038/s41522-019-0099-0. 31552139 PMC6753144

[28] Edmond MB, Ober JF, Dawson JD, Weinbaum DL, Wenzel RP. 1996. Vancomycin-resistant enterococcal bacteremia: Natural history and attributable mortality. Clin Infect Dis 23:1234–1239. doi:10.1093/clinids/23.6.1234. 8953064

[29] Fisher K, Phillips C. 2009. The ecology, epidemiology and virulence of Enterococcus. Microbiology (Reading) 155:1749–1757. doi:10.1099/mic.0.026385-0. 19383684

[30] Flahaut S, Hartke A, Giard J, Benachour A, Boutibonnes P, Auffray Y. 1996. Relationship between stress response towards bile salts, acid and heat treatment in *Enterococcus faecalis*. FEMS Microbiol Lett 138:49–54. doi:10.1111/j.1574-6968.1996.tb08133.x. 8674969

[31] Huycke MM, Sahm DF, Gilmore MS. 1998. Multiple-drug resistant enterococci: The nature of the problem and an agenda for the future. Emerg Infect Dis 4:239–249. doi:10.3201/eid0402.980211. 9621194 PMC2640141

[32] Wang X, He X, Jiang Z, Wang J, Chen X, Liu D, Wang F, Guo Y, Zhao J, Liu F, Huang L, Yuan J. 2010. Proteomic analysis of the *Enterococcus faecalis* V583 strain and clinical isolate V309 under vancomycin treatment. J Proteome Res 9:1772–1785. doi:10.1021/pr901216e. 20128627

[33] Yin W, Wang Y, Liu L, He J. 2019. Biofilms: The microbial “protective clothing” in extreme environments. Int J Mol Sci 20:3423. doi:10.3390/ijms20143423. PMC6679078 31336824

[34] Frank KL, Guiton PS, Barnes AMT, Manias DA, Chuang-Smith ON, Kohler PL, Spaulding AR, Hultgren SJ, Schlievert PM, Dunny GM. 2013. AhrC and Eep are biofilm infection-associated virulence factors in *Enterococcus faecalis*. Infect Immun 81:1696–1708. doi:10.1128/IAI.01210-12. 23460519 PMC3648002

[35] Ch'ng J-H, Chong KKL, Lam LN, Wong JJ, Kline KA. 2019. Biofilm-associated infection by enterococci. Nat Rev Microbiol 17:82–94. doi:10.1038/s41579-018-0107-z. 30337708 PMC13729018

[36] Pisithkul T, Schroeder JW, Trujillo EA, Yeesin P, Stevenson DM, Chaiamarit T, Coon JJ, Wang JD, Amador-Noguez D. 2019. Metabolic remodeling during biofilm development of *Bacillus subtilis*. mBio 10:1–32. doi:10.1128/mBio.00623-19. PMC6529636 31113899

[37] Caro-Astorga J, Frenzel E, Perkins JR, Álvarez-Mena A, de Vicente A, Ranea JAG, Kuipers OP, Romero D. 2020. Biofilm formation displays intrinsic offensive and defensive features of *Bacillus cereus*. NPJ Biofilms Microbiomes 6:3. doi:10.1038/s41522-019-0112-7. 31969984 PMC6962202

[38] Zhu Y, Xiong YQ, Sadykov MR, Fey PD, Lei MG, Lee CY, Bayer AS, Somerville GA. 2009. Tricarboxylic acid cycle-dependent attenuation of *Staphylococcus aureus in vivo* virulence by selective inhibition of amino acid transport. Infect Immun 77:4256–4264. doi:10.1128/IAI.00195-09. 19667045 PMC2747957

[39] Zhu Y, Weiss EC, Otto M, Fey PD, Smeltzer MS, Somerville GA. 2007. *Staphylococcus aureus* biofilm metabolism and the influence of arginine on polysaccharide intercellular adhesin synthesis, biofilm formation, and pathogenesis. Infect Immun 75:4219–4226. doi:10.1128/IAI.00509-07. 17576756 PMC1951195

[40] Seneviratne CJ, Suriyanarayanan T, Swarup S, Chia KHB, Nagarajan N, Zhang C. 2017. Transcriptomics analysis reveals putative genes involved in biofilm formation and biofilm-associated drug resistance of *Enterococcus faecalis*. J Endod 43:949–955. doi:10.1016/j.joen.2017.01.020. 28457636

[41] Manias DA, Dunny GM. 2018. Expression of adhesive pili and the collagen binding adhesin Ace is activated by ArgR family transcription factors in *Enterococcus faecalis*. J Bacteriol 200:1–16. doi:10.1128/JB.00269-18. PMC6112011 29986940

[42] Moormeier DE, Bayles KW. 2017. *Staphylococcus aureus* biofilm: a complex developmental organism. Mol Microbiol 104:365–376. doi:10.1111/mmi.13634. 28142193 PMC5397344

[43] Bowers JR, Driebe EM, Albrecht V, McDougal LK, Granade M, Roe CC, Lemmer D, Rasheed JK, Engelthaler DM, Keim P, Limbago BM. 2018. Improved subtyping of *Staphylococcus aureus* clonal complex 8 strains based on whole-genome phylogenetic analysis. mSphere 3:1–15. doi:10.1128/mSphere.00464-17. PMC5932376 29720527

[44] Paulsen IT, Banerjei L, Myers GSA, Nelson KE, Seshadri R, Read TD, Fouts DE, Eisen JA, Gill SR, Heidelberg JF, Tettelin H, Dodson RJ, Umayam L, Brinkac L, Beanan M, Daugherty S, DeBoy RT, Durkin S, Kolonay J, Madupu R, Nelson W, Vamathevan J, Tran B, Upton J, Hansen T, Shetty J, Khouri H, Utterback T, Radune D, Ketchum KA, Dougherty BA, Fraser CM. 2003. Role of mobile DNA in the evolution of vancomycin-resistant *Enterococcus faecalis*. Science 299:2071–2074. doi:10.1126/science.1080613. 12663927

[45] Kim EB, Kopit LM, Harris LJ, Marco ML. 2012. Draft genome sequence of the quality control strain *Enterococcus faecalis* ATCC 29212. J Bacteriol 194:6006–6007. doi:10.1128/JB.01423-12. 23045510 PMC3486070

[46] Wattam AR, Davis JJ, Assaf R, Boisvert S, Brettin T, Bun C, Conrad N, Dietrich EM, Disz T, Gabbard JL, Gerdes S, Henry CS, Kenyon RW, Machi D, Mao C, Nordberg EK, Olsen GJ, Murphy-Olson DE, Olson R, Overbeek R, Parrello B, Pusch GD, Shukla M, Vonstein V, Warren A, Xia F, Yoo H, Stevens RL. 2017. Improvements to PATRIC, the all-bacterial Bioinformatics Database and Analysis Resource Center. Nucleic Acids Res 45:D535–D542. doi:10.1093/nar/gkw1017. 27899627 PMC5210524

[47] Xavier JC, Patil KR, Rocha I. 2018. Metabolic models and gene essentiality data reveal essential and conserved metabolism in prokaryotes. PLoS Comput Biol 14:e1006556–23. doi:10.1371/journal.pcbi.1006556. 30444863 PMC6283598

[48] Sadovskaya I, Vinogradov E, Flahaut S, Kogan G, Jabbouri S. 2005. Extracellular carbohydrate-containing polymers of a model biofilm-producing strain, *Staphylococcus epidermidis* RP62A. Infect Immun 73:3007–3017. doi:10.1128/IAI.73.5.3007-3017.2005. 15845508 PMC1087347

[49] Sugimoto S, Sato F, Miyakawa R, Chiba A, Onodera S, Hori S, Mizunoe Y. 2018. Broad impact of extracellular DNA on biofilm formation by clinically isolated Methicillin-resistant and -sensitive strains of *Staphylococcus aureus*. Sci Rep 8:2254. doi:10.1038/s41598-018-20485-z. 29396526 PMC5797107

[50] Mohamed JA, Huang DB. 2007. Biofilm formation by enterococci. J Med Microbiol 56:1581–1588. doi:10.1099/jmm.0.47331-0. 18033823

[51] Yan F, Yu Y, Gozzi K, Chen Y, Guo J, Chai Y. 2017. Genome-wide investigation of biofilm formation in *Bacillus cereus*. Appl Environ Microbiol 83:e00561-17. doi:10.1128/AEM.00561-17. 28432092 PMC5478996

[52] Huang DW, Sherman BT, Lempicki RA. 2009. Systematic and integrative analysis of large gene lists using DAVID bioinformatics resources. Nat Protoc 4:44–57. doi:10.1038/nprot.2008.211. 19131956

[53] Karp PD, Latendresse M, Paley SM, Krummenacker M, Ong QD, Billington R, Kothari A, Weaver D, Lee T, Subhraveti P, Spaulding A, Fulcher C, Keseler IM, Caspi R. 2016. Pathway tools version 19.0 update: Software for pathway/genome informatics and systems biology. Brief Bioinform 17:877–890. doi:10.1093/bib/bbv079. 26454094 PMC5036846

[54] Fuchs S, Mehlan H, Bernhardt J, Hennig A, Michalik S, Surmann K, Pané-Farré J, Giese A, Weiss S, Backert L, Herbig A, Nieselt K, Hecker M, Völker U, Mäder U. 2018. Aureo Wiki: The repository of the *Staphylococcus aureus* research and annotation community. Int J Med Microbiol 308:558–568. doi:10.1016/j.ijmm.2017.11.011. 29198880

[55] Resch A, Rosenstein R, Nerz C, Götz F. 2005. Differential gene expression profiling of Staphylococcus aureus cultivated under biofilm and planktonic conditions. Appl Environ Microbiol 71:2663–2676. doi:10.1128/AEM.71.5.2663-2676.2005. 15870358 PMC1087559

[56] Tomlinson BR, Malof ME, Lindsey Shaw N. 2020. A global transcriptomic analysis of 2 *Staphylococcus aureus* biofilm formation across diverse clonal lineages. Microbial Genomics 7:e000598. 10.1099/mgen.0.000598 PMC8477394 34227933

[57] Suryaletha K, Narendrakumar L, John J, Radhakrishnan MP, George S, Thomas S. 2019. Decoding the proteomic changes involved in the biofilm formation of *Enterococcus faecalis* SK460 to elucidate potential biofilm determinants. BMC Microbiol 19:146. doi:10.1186/s12866-019-1527-2. 31253082 PMC6599329

[58] Kilstrup M, Hammer K, Ruhdal Jensen P, Martinussen J. 2005. Nucleotide metabolism and its control in lactic acid bacteria. FEMS Microbiol Rev 29:555–590. doi:10.1016/j.femsre.2005.04.006. 15935511

[59] Fey PD, Endres JL, Yajjala VK, Widhelm TJ, Boissy RJ, Bose JL, Bayles KW. 2013. A Genetic resource for rapid and comprehensive phenotype screening of nonessential *Staphylococcus aureus* genes. mBio 4:e00537-12. doi:10.1128/mBio.00537-12. 23404398 PMC3573662

[60] Zhang Y, Morar M, Ealick SE. 2008. Structural biology of the purine biosynthetic pathway. Cell Mol Life Sci 65:3699–3724. doi:10.1007/s00018-008-8295-8. 18712276 PMC2596281

[61] Spurr IB, Birts CN, Cuda F, Benkovic SJ, Blaydes JP, Tavassoli A. 2012. Small Molecule Inhibitor of AICAR Transformylase Homodimerization. Chembiochem 13:1628–1634. doi:10.1002/cbic.201200279. 22764122 PMC3517147

[62] Malheiro J, Simões M. 2016. Antimicrobial resistance of biofilms in medical devices, p 98–113. *In* Ying Deng, Wei Lv, Biofilms and implantable medical devices: Infection and control. Elsevier Inc, United Kingdom.

[63] Floyd KA, Eberly AR, Hadjifrangiskou M. 2017. Adhesion of bacteria to surfaces and biofilm formation on medical devices, p 47–95. *In* Ying Deng, Wei Lv, Biofilms and implantable medical devices. Elsevier, United Kingdom.

[64] Tendolkar PM, Baghdayan AS, Gilmore MS, Shankar N. 2004. Enterococcal surface protein, Esp, enhances biofilm formation by *Enterococcus faecalis*. Infect Immun 72:6032–6039. doi:10.1128/IAI.72.10.6032-6039.2004. 15385507 PMC517584

[65] Willett JLE, Dale JL, Kwiatkowski LM, Powers JL, Korir ML, Kohli R, Barnes AMT, Dunny GM. 2021. Comparative biofilm assays using *Enterococcus faecalis* OG1RF identify new determinants of biofilm formation. mBio 12:e0101121. doi:10.1128/mBio.01011-21. 34126766 PMC8262879

[66] Dogsa I, Brloznik M, Stopar D, Mandic-Mulec I. 2013. Exopolymer diversity and the role of levan in *Bacillus subtilis* biofilms. PLoS One 8:e62044. doi:10.1371/journal.pone.0062044. 23637960 PMC3637382

[67] Stipetic LH, Dalby MJ, Davies RL, Morton FR, Ramage G, Burgess KEV. 2016. A novel metabolomic approach used for the comparison of *Staphylococcus aureus* planktonic cells and biofilm samples. Metabolomics 12:75. doi:10.1007/s11306-016-1002-0. 27013931 PMC4783440

[68] Ammons MCB, Tripet BP, Carlson RP, Kirker KR, Gross MA, Stanisich JJ, Copié V. 2014. Quantitative NMR metabolite profiling of methicillin-resistant and methicillin-susceptible *Staphylococcus aureus* discriminates between biofilm and planktonic phenotypes. J Proteome Res 13:2973–2985. doi:10.1021/pr500120c. 24809402 PMC4059261

[69] Resch A, Leicht S, Saric M, Pásztor L, Jakob A, Götz F, Nordheim A. 2006. Comparative proteome analysis of *Staphylococcus aureus* biofilm and planktonic cells and correlation with transcriptome profiling. PROTEOMICS 6:1867–1877. doi:10.1002/pmic.200500531. 16470655

[70] Liu J, Yang L, Hou Y, Soteyome T, Zeng B, Su J, Li L, Li B, Chen D, Li Y, Wu A, Shirtliff ME, Harro JM, Xu Z, Peters BM. 2018. Transcriptomics study on *Staphylococcus aureus* biofilm under low concentration of ampicillin. Front Microbiol 9:2413. doi:10.3389/fmicb.2018.02413. 30425687 PMC6218852

[71] Philips J, Rabaey K, Lovley DR, Vargas M. 2017. Biofilm formation by clostridium ljungdahlii is induced by sodium chloride stress: Experimental evaluation and transcriptome analysis. PLoS One 12:e0170406–25. doi:10.1371/journal.pone.0170406. 28118386 PMC5261816

[72] Kim JK, Kwon JY, Kim SK, Han SH, Won YJ, Lee JH, Kim CH, Fukatsu T, Lee BL. 2014. Purine biosynthesis, biofilm formation, and persistence of an insect-microbe gut symbiosis. Appl Environ Microbiol 80:4374–4382. doi:10.1128/AEM.00739-14. 24814787 PMC4068690

[73] Shaffer CL, Zhang EW, Dudley AG, Dixon BREA, Guckes KR, Breland EJ, Floyd KA, Casella DP, Algood HMS, Clayton DB, Hadjifrangiskou M. 2017. Purine biosynthesis metabolically constrains intracellular survival of uropathogenic *Escherichia coli*. Infect Immun 85:e00471-1. doi:10.1128/IAI.00471-16. PMC5203662 27795353

[74] Yoshioka S, Newell PD. 2016. Disruption of de novo purine biosynthesis in *Pseudomonas fluorescens* Pf0-1 leads to reduced biofilm formation and a reduction in cell size of surface-attached but not planktonic cells. PeerJ 2016:1–24. 10.7717/peerj.1543 PMC4715448 26788425

[75] Ge X, Kitten T, Chen Z, Lee SP, Munro CL, Xu P. 2008. Identification of *Streptococcus sanguinis* genes required for biofilm formation and examination of their role in endocarditis virulence. Infect Immun 76:2551–2559. doi:10.1128/IAI.00338-08. 18390999 PMC2423065

[76] Dengler V, Foulston L, DeFrancesco AS, Losick R. 2015. An electrostatic net model for the role of extracellular DNA in biofilm formation by *Staphylococcus aureus*. J Bacteriol 197:3779–3787. doi:10.1128/JB.00726-15. 26416831 PMC4652055

[77] Thomas VC, Thurlow LR, Boyle D, Hancock LE. 2008. Regulation of autolysis-dependent extracellular DNA release by *Enterococcus faecalis* extracellular proteases influences biofilm development. J Bacteriol 190:5690–5698. doi:10.1128/JB.00314-08. 18556793 PMC2519388

[78] Vilain S, Pretorius JM, Theron J, Brözel VS. 2009. DNA as an adhesin: *Bacillus cereus* requires extracellular DNA to form biofilms. Appl Environ Microbiol 75:2861–2868. doi:10.1128/AEM.01317-08. 19251901 PMC2681682

[79] Goncheva MI, Flannagan RS, Heinrichs DE. 2020. De novo purine biosynthesis is required for intracellular growth of *Staphylococcus aureus* and for the hypervirulence phenotype of a pure mutant. Infection and Immunity 88:1–15. 10.1128/IAI.00104-20 PMC7171247 32094249

[80] Goncheva MI, Flannagan RS, Sterling BE, Laakso HA, Friedrich NC, Kaiser JC, Watson DW, Wilson CH, Sheldon JR, McGavin MJ, Kiser PK, Heinrichs DE. 2019. Stress-induced inactivation of the *Staphylococcus aureus* purine biosynthesis repressor leads to hypervirulence. Nat Commun 10:775. doi:10.1038/s41467-019-08724-x. 30770821 PMC6377658

[81] Li L, Abdelhady W, Donegan NP, Seidl K, Cheung A, Zhou YF, Yeaman MR, Bayer AS, Xiong YQ. 2018. Role of purine biosynthesis in persistent methicillin-resistant *Staphylococcus aureus* infection. J Infect Dis 218:1367–1377. doi:10.1093/infdis/jiy340. 29868791 PMC6151072

[82] Bose JL, Fey PD, Bayles KW. 2013. Genetic tools to enhance the study of gene function and regulation in *Staphylococcus aureus*. Appl Environ Microbiol 79:2218–2224. doi:10.1128/AEM.00136-13. 23354696 PMC3623228

[83] O'Toole GA. 2011. Microtiter dish biofilm formation assay. J Vis Exp 47:2437. doi:10.3791/2437. PMC3182663 21307833

[84] Langmead B, Salzberg SL. 2012. Fast gapped-read alignment with Bowtie 2. Nat Methods 9:357–359. doi:10.1038/nmeth.1923. 22388286 PMC3322381

[85] Herbert S, Ziebandt AK, Ohlsen K, Schäfer T, Hecker M, Albrecht D, Novick R, Götz F. 2010. Repair of global regulators in *Staphylococcus aureus* 8325 and comparative analysis with other clinical isolates. Infect Immun 78:2877–2889. doi:10.1128/IAI.00088-10. 20212089 PMC2876537

[86] Love MI, Huber W, Anders S. 2014. Moderated estimation of fold change and dispersion for RNA-seq data with DESeq2. Genome Biol 15:550. doi:10.1186/s13059-014-0550-8. 25516281 PMC4302049

