# Supplementary material for: The de novo Purine Biosynthesis Pathway Is the Only Commonly Regulated Cellular Pathway during Biofilm Formation in TSB-Based Medium in Staphylococcus aureus and Enterococcus faecalis
Source: Microbiol Spectr. 2021 Dec 22;9(3):e00804-21. doi: 10.1128/Spectrum.00804-21 (PMC8693917; doi:10.1128/Spectrum.00804-21)
Supplement: SUPPLEMENTAL FILE 4 — Supplemental material. Download SPECTRUM00804-21_Supp_1_seq10.pdf, PDF file, 3.7 MB [file spectrum00804-21_supp_1_seq10.pdf]

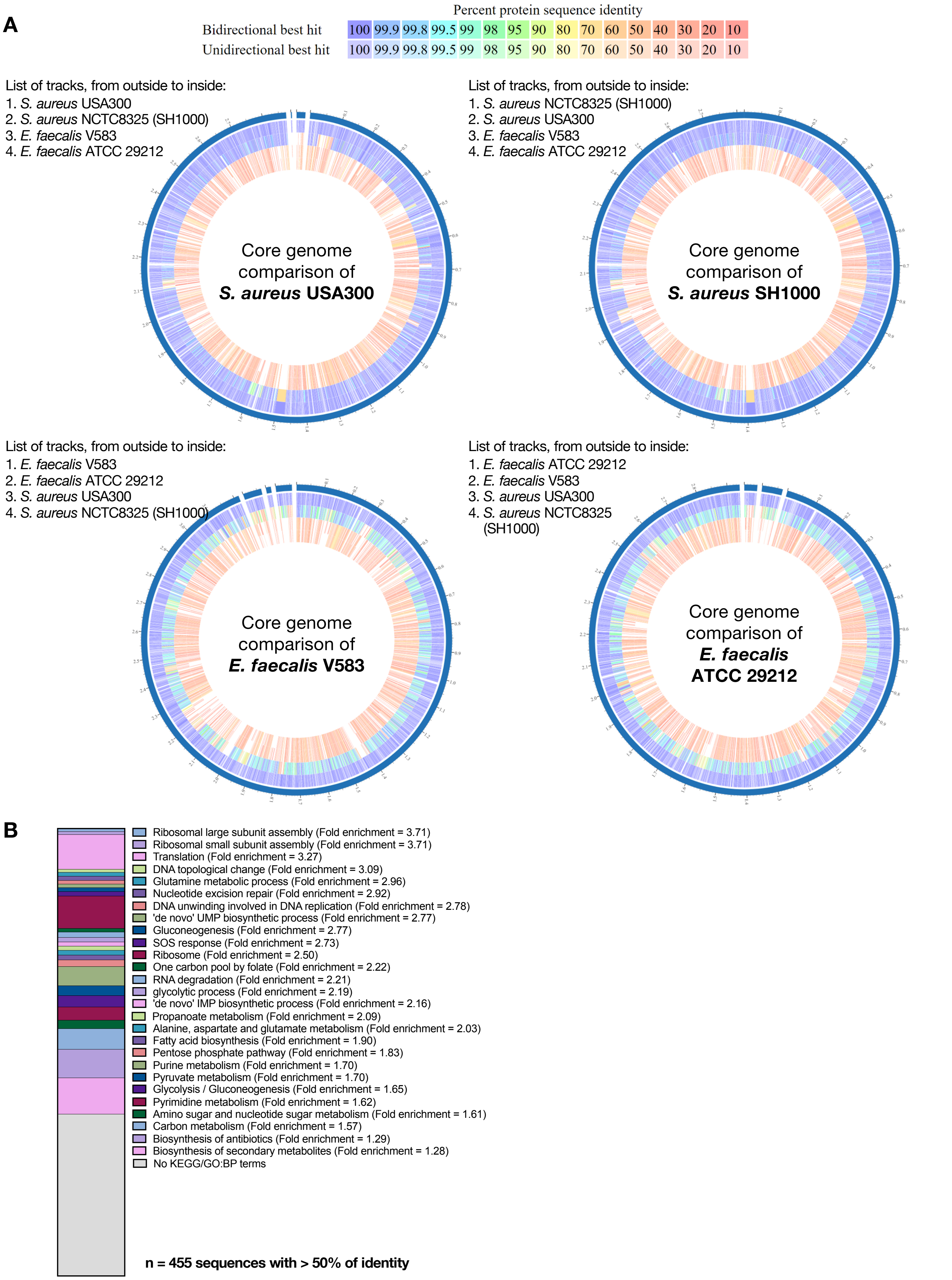
A
Percent protein sequence identity
Bidirectional best hit 100 99.9 99.8 99.5 99 98 95 90 80 70 60 50 40 30 20 10
Unidirectional best hit 100 99.9 99.8 99.5 99 98 95 90 80 70 60 50 40 30 20 10
List of tracks, from outside to inside:
1. *S. aureus* USA300
2. *S. aureus* NCTC8325 (SH1000)
3. *E. faecalis* V583
4. *E. faecalis* ATCC 29212
Core genome comparison of **S. aureus USA300**
List of tracks, from outside to inside:
1. *S. aureus* NCTC8325 (SH1000)
2. *S. aureus* USA300
3. *E. faecalis* V583
4. *E. faecalis* ATCC 29212
Core genome comparison of **S. aureus SH1000**
List of tracks, from outside to inside:
1. *E. faecalis* V583
2. *E. faecalis* ATCC 29212
3. *S. aureus* USA300
4. *S. aureus* NCTC8325 (SH1000)
Core genome comparison of **E. faecalis V583**
List of tracks, from outside to inside:
1. *E. faecalis* ATCC 29212
2. *E. faecalis* V583
3. *S. aureus* USA300
4. *S. aureus* NCTC8325 (SH1000)
Core genome comparison of **E. faecalis ATCC 29212**
B
Ribosomal large subunit assembly (Fold enrichment = 3.71)
Ribosomal small subunit assembly (Fold enrichment = 3.71)
Translation (Fold enrichment = 3.27)
DNA topological change (Fold enrichment = 3.09)
Glutamine metabolic process (Fold enrichment = 2.96)
Nucleotide excision repair (Fold enrichment = 2.92)
DNA unwinding involved in DNA replication (Fold enrichment = 2.78)
'de novo' UMP biosynthetic process (Fold enrichment = 2.77)
Gluconeogenesis (Fold enrichment = 2.77)
SOS response (Fold enrichment = 2.73)
Ribosome (Fold enrichment = 2.50)
One carbon pool by folate (Fold enrichment = 2.22)
RNA degradation (Fold enrichment = 2.21)
glycolytic process (Fold enrichment = 2.19)
'de novo' IMP biosynthetic process (Fold enrichment = 2.16)
Propanoate metabolism (Fold enrichment = 2.09)
Alanine, aspartate and glutamate metabolism (Fold enrichment = 2.03)
Fatty acid biosynthesis (Fold enrichment = 1.90)
Pentose phosphate pathway (Fold enrichment = 1.83)
Purine metabolism (Fold enrichment = 1.70)
Pyruvate metabolism (Fold enrichment = 1.70)
Glycolysis / Gluconeogenesis (Fold enrichment = 1.65)
Pyrimidine metabolism (Fold enrichment = 1.62)
Amino sugar and nucleotide sugar metabolism (Fold enrichment = 1.61)
Carbon metabolism (Fold enrichment = 1.57)
Biosynthesis of antibiotics (Fold enrichment = 1.29)
Biosynthesis of secondary metabolites (Fold enrichment = 1.28)
No KEGG/GO:BP terms
n = 455 sequences with > 50% of identity

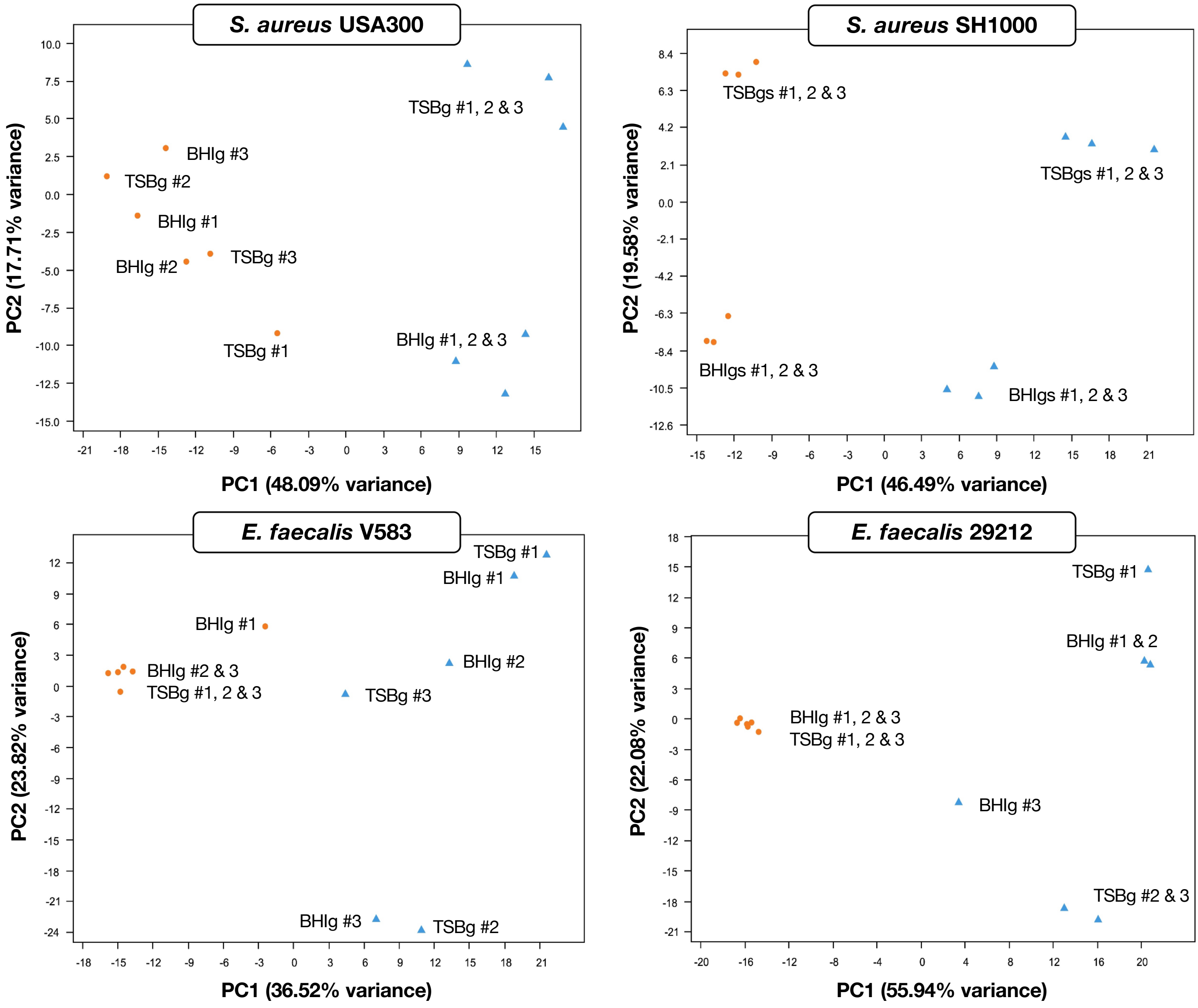

FIG S2. Principal component analysis (PCA) of the variance between the biological replicates from the transcriptomic analysis. The blue triangles represent planktonic cultures while the orange circles represent biofilm cultures of *S. aureus* or *E. faecalis*. The medium and the number of the biological replicate are indicated next to the geometrical icon.

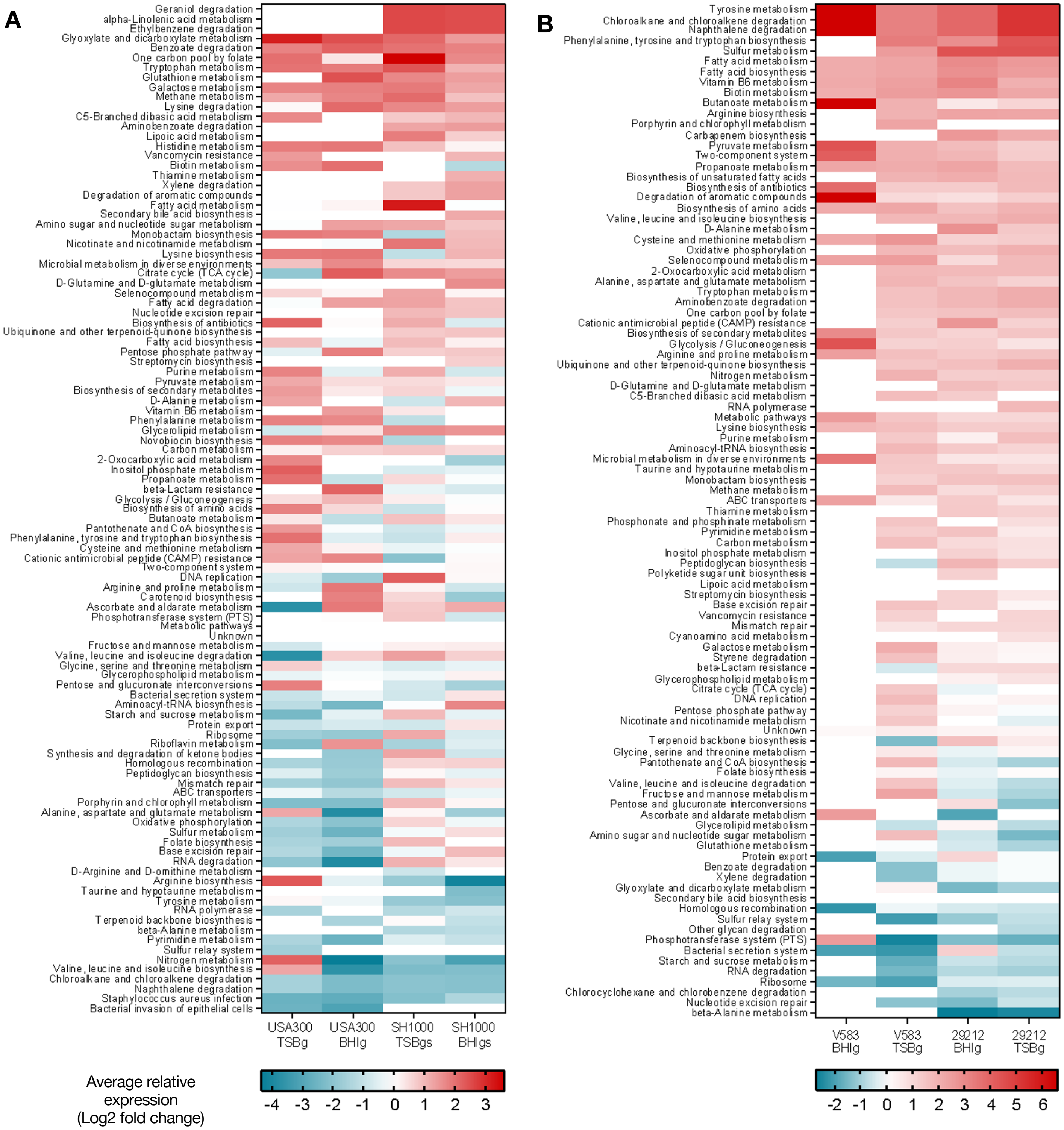

FIG S3. Relative expression of KEGG terms during biofilm formation. Each row of both heatmaps represents a single functional annotation term from DAVID Bioinformatic software using KEGG pathways annotation source. The term's relative expression is the average of the log2 fold change of every associated gene with an adjusted *P*-value ≤ 0.05 from the transcriptomic analysis. The red (upregulated) and blue (downregulated) colour scale indicates relative expression level in average log2 fold change. (A) Functional annotation terms' (total = 103) relative expression in *S. aureus* USA300 and SH1000, in BHIg(s) or TSBg(s) medium. (B) Functional annotation terms' (total = 97) relative expression in *E. faecalis* V583 and 29212, in BHIg or TSBg medium.

	***S. aureus* in BHIg(s)**	***S. aureus* in TSBg(s)**	***E. faecalis* in BHIg**	***E. faecalis* in TSBg**
1	formate catabolic process	formate catabolic process	alcohol metabolic process	shikimate metabolic process
2	serine family amino acid biosynthetic process	purine nucleobase biosynthetic process	carbon utilization	tyrosine biosynthetic process
3	hemolysis by symbiont of host erythrocytes	'de novo' IMP biosynthetic process	programmed cell death	alcohol metabolic process
4	chromosome condensation	adenine biosynthetic process	cytolysis	carbon utilization
5	regulation of nucleic acid-templated transcription	ribonucleoside monophosphate biosynthetic process	thiamine biosynthetic process	aromatic amino acid family biosynthetic process
6	fatty acid beta-oxidation using acyl-CoA dehydrogenase	purine nucleotide biosynthetic process	fatty acid elongation	chorismate biosynthetic process
7	lipid homeostasis	IMP metabolic process	protein homotetramerization	cellular amino acid biosynthetic process
8	potassium ion transmembrane transport	serine family amino acid biosynthetic process	carboxylic acid metabolic process	L-phenylalanine biosynthetic process
9	L-lysine catabolic process to acetyl-CoA via saccharopine	'de novo' AMP biosynthetic process	fatty acid biosynthetic process	cysteine biosynthetic process from serine
10	programmed cell death	glutamine metabolic process	S-adenosylmethionine biosynthetic process	fatty acid elongation
11	lipid catabolic process	fatty acid beta-oxidation using acyl-CoA dehydrogenase	protein flavinylation	protein homotetramerization
12	folic acid-containing compound biosynthetic process	lipid homeostasis	oligopeptide transport	purine ribonucleoside salvage
13	tetrahydrofolate interconversion	folic acid-containing compound biosynthetic process	quorum sensing	XMP salvage
14	glyoxylate cycle	tetrahydrofolate interconversion	one-carbon metabolic process	xanthine metabolic process
15	tricarboxylic acid cycle	polysaccharide biosynthetic process	biosynthetic process	S-adenosylmethionine biosynthetic process
1	arginine catabolic process to ornithine	negative regulation of blood coagulation in other organism	SOS response	glycine betaine transport
2	arginine catabolic process	cytolysis in other organism	tRNA wobble adenosine to inosine editing	protein processing
3	arginine deiminase pathway	defense response to bacterium	ribosomal small subunit biogenesis	allantoin catabolic process
4	carbamoyl phosphate catabolic process	threonine catabolic process	DNA-templated transcription, termination	purine nucleobase metabolic process
5	L-serine catabolic process	L-serine catabolic process	transcription antitermination	protein refolding
6	L-threonine catabolic process to propionate	L-threonine catabolic process to propionate	phosphorelay signal transduction system	response to heat
7	negative regulation of blood coagulation in other organism	threonyl-tRNA aminoacylation	DNA-templated transcription, elongation	glycerol catabolic process
8	arginine biosynthetic process via ornithine	glycine betaine biosynthetic process from choline	regulation of DNA-templated transcription, elongation	phosphoenolpyruvate-dependent sugar phosphotransferase system
9	pyrimidine ribonucleotide biosynthetic process	aspartate transport	transmembrane transport	Mo-molybdopterin cofactor biosynthetic process
10	threonine catabolic process	L-glutamate transport	DNA repair	DNA-dependent DNA replication
11	urea cycle	barrier septum assembly	translation	glycerol-3-phosphate metabolic process
12	fatty acid metabolic process	cytolysis		methionine transport
13	cytolysis in other organism	glutamate biosynthetic process		amino acid transport
14	DNA-templated transcription, termination	RNA modification		barrier septum assembly
15	'de novo' pyrimidine nucleobase biosynthetic process	riboflavin biosynthetic process		SOS response

Figure S4. Average relative expression of GO:BP terms by species during biofilm formation Table of the top 15 upregulated and downregulated GO:BP terms for each species and by media, by calculating the average of the relative expression of each term for the 2 strains. Because of the low number of DEGs for *E. faecalis*, only 11 terms were consistently downregulated in BHIg.

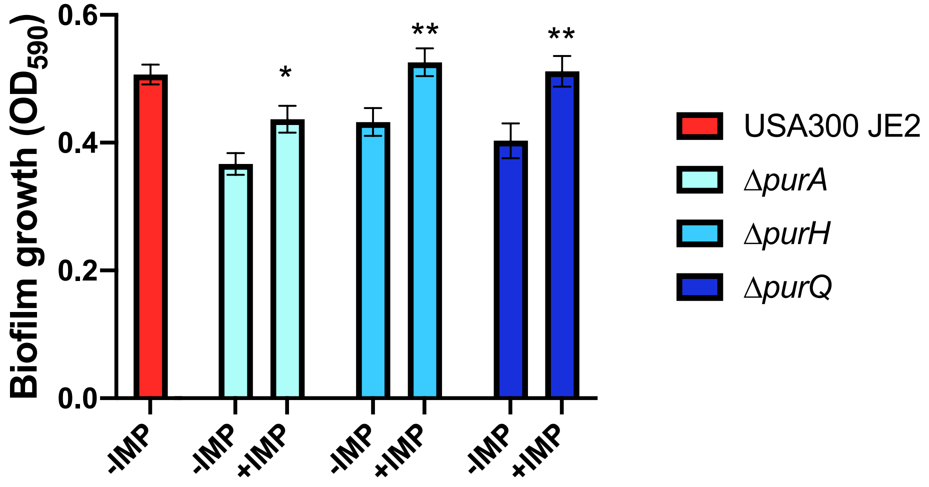

Figure S5. Addition of IMP restores biofilm formation in *de novo* purine mutants. Biofilms of *S. aureus* USA300 containing transposon insertion (NTML) in gene encoding for the purine biosynthesis pathway were formed in TSBg, with or without IMP (1 mg/mL) and quantified by crystal violet. Each bar represents biofilm production (in OD_{590}) of 18 replicates ± SEM (Student t-test; * *P*-value < 0.05; ** *P*-value < 0.01).

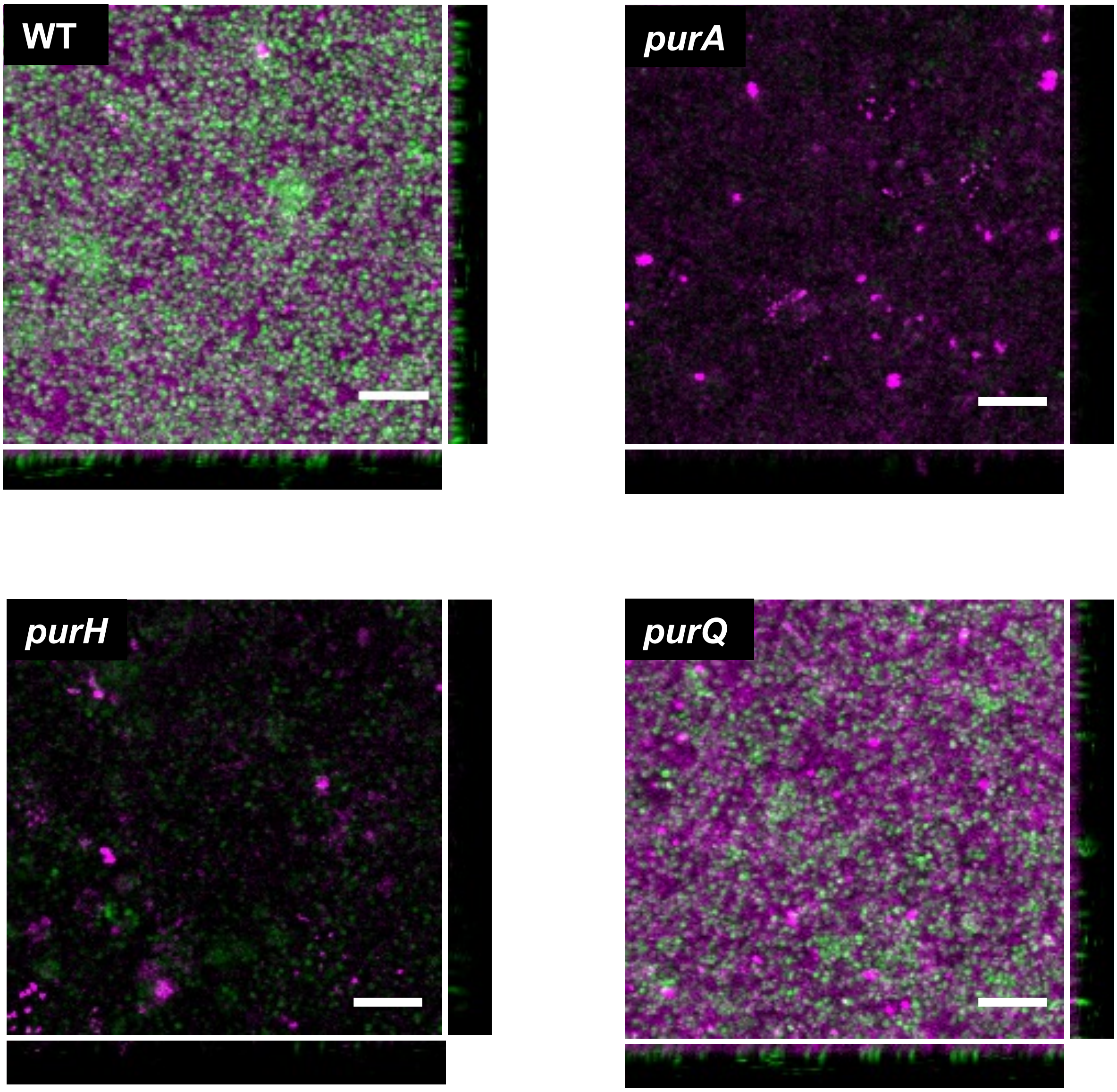

Figure S6. Inhibition of *de novo* purine biosynthesis pathway affects biofilm formation. Biofilms of *S. aureus* USA300 containing transposon insertion (NTML) in gene encoding for the purine biosynthesis pathway were formed in TSBg for 24 h, and examined by confocal microscopy. Biofilms were stained with Concanavalin A Alexa Fluor^{TM} 647 conjugate (pink) for exopolysaccharides, and Syto9 (green) for cells. Images are representative of 2 biological replicates, and scale bar is 10 µm.

Table S1. Strains used in this study

Organisme	Strain name	Genotype	Provenance
E. faecalis	V583	Wild type	Lab stock[1]
E. faecalis	ATCC 29212	Wild type	Lab stock[1]
S. aureus	USA300	Wild type	Lab stock[1]
S. aureus	USA300 JE2	Wild type	Lab stock[2]
S. aureus	SH1000	Wild type	Lab stock[1]
S. aureus	NE191	USA300 *folD*	NTML[2]
S. aureus	NE353	USA300 *purH*	NTML[2]
S. aureus	NE494	USA300 *purQ*	NTML[2]
S. aureus	NE529	USA300 *purA*	NTML[2]
S. aureus	NE750	USA300 *purD*	NTML[2]
S. aureus	NE1101	USA300 *purM*	NTML[2]
S. aureus	NE1785	USA300 *purN*	NTML[2]

[1] These strains come from the Malouin lab stock.

[2] These strains are kind gifts from the JP Côté lab stock (Université de Sherbrooke). Reference: Bose JL, Fey PD, Bayles KW. 2013. Genetic tools to enhance the study of gene function and regulation in *Staphylococcus aureus*. Appl Environ Microbiol 79:2218–2224.

Table S2. Prokaryotic Illumina libraries primers.

Primers name	Sequence (5' – 3')	Usage
TruSeq-Mlpt-YIGA-bot	/5Phos/GAT CGG AAG AGC ACA CGT CT	Adaptor
YIGA-top	ACA CTC TTT CCC TAC ACG ACG CTC TTC CGA TC*T	Adaptor
IGA-PE-F primer	AATGATACGGCGACCACCGAGATCTACAC TAGATCGC ACACTCTTTCCCTACACGACGCT	PCR amplification
I-Tru-Index-R01	CAAGCAGAAGACGGCATACGAGAT ATTGGTCA GTGACTGGAGTTCAGACGTGTGCTCTTC	PCR amplification
I-Tru-Index-R02	CAAGCAGAAGACGGCATACGAGAT TAAAAATG GTGACTGGAGTTCAGACGTGTGCTCTTC	PCR amplification
I-Tru-Index-R03	CAAGCAGAAGACGGCATACGAGAT ATCACTGT GTGACTGGAGTTCAGACGTGTGCTCTTC	PCR amplification
I-Tru-Index-R04	CAAGCAGAAGACGGCATACGAGAT TATTTCAC GTGACTGGAGTTCAGACGTGTGCTCTTC	PCR amplification
I-Tru-Index-R05	CAAGCAGAAGACGGCATACGAGAT ATATTGGC GTGACTGGAGTTCAGACGTGTGCTCTTC	PCR amplification
I-Tru-Index-R06	CAAGCAGAAGACGGCATACGAGAT TATACAAG GTGACTGGAGTTCAGACGTGTGCTCTTC	PCR amplification
I-Tru-Index-R07	CAAGCAGAAGACGGCATACGAGAT ATGATCTG GTGACTGGAGTTCAGACGTGTGCTCTTC	PCR amplification
I-Tru-Index-R08	CAAGCAGAAGACGGCATACGAGAT TACTCTAC GTGACTGGAGTTCAGACGTGTGCTCTTC	PCR amplification
I-Tru-Index-R09	CAAGCAGAAGACGGCATACGAGAT ATAAGCTA GTGACTGGAGTTCAGACGTGTGCTCTTC	PCR amplification
I-Tru-Index-R10	CAAGCAGAAGACGGCATACGAGAT TAGTATAG GTGACTGGAGTTCAGACGTGTGCTCTTC	PCR amplification
I-Tru-Index-R11	CAAGCAGAAGACGGCATACGAGAT ATTACAAG GTGACTGGAGTTCAGACGTGTGCTCTTC	PCR amplification
I-Tru-Index-R12	CAAGCAGAAGACGGCATACGAGAT TAATTGGC GTGACTGGAGTTCAGACGTGTGCTCTTC	PCR amplification
I-Tru-Index-R13	CAAGCAGAAGACGGCATACGAGAT ATCTCTAC GTGACTGGAGTTCAGACGTGTGCTCTTC	PCR amplification
I-Tru-Index-R14	CAAGCAGAAGACGGCATACGAGAT TAGATCTG GTGACTGGAGTTCAGACGTGTGCTCTTC	PCR amplification
I-Tru-Index-R15	CAAGCAGAAGACGGCATACGAGAT ATGCGGAC GTGACTGGAGTTCAGACGTGTGCTCTTC	PCR amplification
I-Tru-Index-R16	CAAGCAGAAGACGGCATACGAGAT TAATCAGT GTGACTGGAGTTCAGACGTGTGCTCTTC	PCR amplification
I-Tru-Index-R17	CAAGCAGAAGACGGCATACGAGAT ATTTTCAC GTGACTGGAGTTCAGACGTGTGCTCTTC	PCR amplification
I-Tru-Index-R18	CAAGCAGAAGACGGCATACGAGAT TACACTGT GTGACTGGAGTTCAGACGTGTGCTCTTC	PCR amplification
I-Tru-Index-R19	CAAGCAGAAGACGGCATACGAGAT ATGGCCAC GTGACTGGAGTTCAGACGTGTGCTCTTC	PCR amplification
I-Tru-Index-R20	CAAGCAGAAGACGGCATACGAGAT TACCGGTG GTGACTGGAGTTCAGACGTGTGCTCTTC	PCR amplification
I-Tru-Index-R21	CAAGCAGAAGACGGCATACGAGAT ATCGAAAC GTGACTGGAGTTCAGACGTGTGCTCTTC	PCR amplification

I-Tru-Index-R22	CAAGCAGAAGACGGCATACGAGAT TATAGTTG GTGACTGGAGTTCAGACGTGTGCTCTTC	PCR amplification
I-Tru-Index-R23	CAAGCAGAAGACGGCATACGAGAT ATCGTACG GTGACTGGAGTTCAGACGTGTGCTCTTC	PCR amplification
I-Tru-Index-R24	CAAGCAGAAGACGGCATACGAGAT TAGAATGA GTGACTGGAGTTCAGACGTGTGCTCTTC	PCR amplification
I-Tru-Index-R25	CAAGCAGAAGACGGCATACGAGAT ATGCTACC GTGACTGGAGTTCAGACGTGTGCTCTTC	PCR amplification
I-Tru-Index-R26	CAAGCAGAAGACGGCATACGAGAT TAATCGTG GTGACTGGAGTTCAGACGTGTGCTCTTC	PCR amplification
I-Tru-Index-R27	CAAGCAGAAGACGGCATACGAGAT ATATCAGT GTGACTGGAGTTCAGACGTGTGCTCTTC	PCR amplification
I-Tru-Index-R28	CAAGCAGAAGACGGCATACGAGAT TAGCGGAC GTGACTGGAGTTCAGACGTGTGCTCTTC	PCR amplification
I-Tru-Index-R29	CAAGCAGAAGACGGCATACGAGAT ATGCTCAT GTGACTGGAGTTCAGACGTGTGCTCTTC	PCR amplification
I-Tru-Index-R30	CAAGCAGAAGACGGCATACGAGAT TACGATTA GTGACTGGAGTTCAGACGTGTGCTCTTC	PCR amplification
I-Tru-Index-R31	CAAGCAGAAGACGGCATACGAGAT ATAGGAAT GTGACTGGAGTTCAGACGTGTGCTCTTC	PCR amplification
I-Tru-Index-R32	CAAGCAGAAGACGGCATACGAGAT ATTAGTTG GTGACTGGAGTTCAGACGTGTGCTCTTC	PCR amplification
I-Tru-Index-R33	CAAGCAGAAGACGGCATACGAGAT TACGAAAC GTGACTGGAGTTCAGACGTGTGCTCTTC	PCR amplification
I-Tru-Index-R34	CAAGCAGAAGACGGCATACGAGAT ATCCGGTG GTGACTGGAGTTCAGACGTGTGCTCTTC	PCR amplification
I-Tru-Index-R35	CAAGCAGAAGACGGCATACGAGAT TAGGCCAC GTGACTGGAGTTCAGACGTGTGCTCTTC	PCR amplification
I-Tru-Index-R36	CAAGCAGAAGACGGCATACGAGAT ATATCGTG GTGACTGGAGTTCAGACGTGTGCTCTTC	PCR amplification
I-Tru-Index-R37	CAAGCAGAAGACGGCATACGAGAT TAGCTACC GTGACTGGAGTTCAGACGTGTGCTCTTC	PCR amplification
I-Tru-Index-R38	CAAGCAGAAGACGGCATACGAGAT ATCGCCTG GTGACTGGAGTTCAGACGTGTGCTCTTC	PCR amplification
I-Tru-Index-R39	CAAGCAGAAGACGGCATACGAGAT ATAAAATG GTGACTGGAGTTCAGACGTGTGCTCTTC	PCR amplification
I-Tru-Index-R40	CAAGCAGAAGACGGCATACGAGAT TATGGTCA GTGACTGGAGTTCAGACGTGTGCTCTTC	PCR amplification
I-Tru-Index-R41	CAAGCAGAAGACGGCATACGAGAT ATATTCCG GTGACTGGAGTTCAGACGTGTGCTCTTC	PCR amplification
I-Tru-Index-R42	CAAGCAGAAGACGGCATACGAGAT ATGTATAG GTGACTGGAGTTCAGACGTGTGCTCTTC	PCR amplification
I-Tru-Index-R43	CAAGCAGAAGACGGCATACGAGAT TAAAGCTA GTGACTGGAGTTCAGACGTGTGCTCTTC	PCR amplification
I-Tru-Index-R44	CAAGCAGAAGACGGCATACGAGAT ATCGATTA GTGACTGGAGTTCAGACGTGTGCTCTTC	PCR amplification
I-Tru-Index-R45	CAAGCAGAAGACGGCATACGAGAT TAGCTCAT GTGACTGGAGTTCAGACGTGTGCTCTTC	PCR amplification
I-Tru-Index-R46	CAAGCAGAAGACGGCATACGAGAT ATGAATGA GTGACTGGAGTTCAGACGTGTGCTCTTC	PCR amplification
I-Tru-Index-R47	CAAGCAGAAGACGGCATACGAGAT TACGTACG GTGACTGGAGTTCAGACGTGTGCTCTTC	PCR amplification
I-Tru-Index-R48	CAAGCAGAAGACGGCATACGAGAT ATCGTGAT GTGACTGGAGTTCAGACGTGTGCTCTTC	PCR amplification

Table S6. Abbreviations used in the purine biosynthesis pathway.

Abbreviation	Nomenclature
(p)ppGpp	guanosine pentophosphate
ADP	adenosine diphosphate
AICAR	5'-aminoimidazole-4-carboxamide ribonucleotide
AIR	5'-aminoimidazole ribotide
AMP	adenosine monophosphate
ATP	adenosine triphosphate
CAIR	5'-phosphoribosyl-4-carboxy-5-aminoimidazol
FAICAR	5'-formamidoimidazole-4-carboxamide ribotide
FGAM	5'-phosphoribosylformylglycinamidine
FGAR	5'-phosphoribosyl-N-formylglycineamid
GAR	glycineamide ribonucleotide
GDP	guanosine diphosphate
GMP	guanosine monophosophate
GTP	guanosine triphosphate
IMP	inosine-5-phosphate
PRA	5'-phosphoribosylamine
PRPP	5'-phosphoribosylamine-1-pyrophosphate
R5P	ribose-5-phosphate
SAICAR	phosphoribosylaminoimidazolesuccinocarboxamide
XMP	xanthosine monophosphate

Figure S1. Core genome analysis between Gram-positive strains in a protein sequence-based homology manner. (A) Circular representation of *S. aureus* and *E. faecalis* strains genomic comparison. Each ring represents the chromosome of a strain, where the outer ring is the reference genome and the three inner rings are the chromosomes of comparison, as indicated on the upper-left corner of each plot. Each line represents a CDS and its % of identity with the reference genome is represented by the blue (high) to the red (low) colour scale. Plots were generated using CIRCOS circular genome data visualization with PATRIC's proteome comparison service. (B) Cellular functions from the genes sharing at least 50% of identity (n = 455) in their protein sequence between the 4 strains of the transcriptomic study, obtained with **PATRIC's proteome comparison service**. Each row from the graph represents the proportion of genes attributed to a function relative to the total number of genes. The fold enrichment of each term is indicated next to its name. Functional annotation was performed with DAVID Bioinformatic software using GO:BP and KEGG pathways as annotation sources.

Figure S2 Principal component analysis (PCA) of the variance between the biological replicates from the transcriptomic analysis. The blue triangles represent planktonic cultures while the orange circles represent biofilm cultures

of *S. aureus* or *E. faecalis*. The medium and the number of the biological replicate are indicated next to the geometrical icon.

Figure S3 Relative expression of KEGG terms during biofilm formation. Each row of both heatmaps represents a single functional annotation term from DAVID Bioinformatic software using KEGG pathways annotation source. The term's relative expression is the average of the log2 fold change of every associated gene with an adjusted *P*-value ≤ 0.05 from the transcriptomic analysis. The red (up-regulated) and blue (down-regulated) colour scale indicates relative expression level in average log2 fold change. (A) Functional annotation terms' (total = 103) relative expression in *S. aureus* USA300 and SH1000, in BHIg(s) or TSBg(s) medium. (B) Functional annotation terms' (total = 97) relative expression in *E. faecalis* V583 and 29212, in BHIg or TSBg medium.

Figure S4. Average relative expression of GO:BP terms by species during biofilm formation. Table of the top 15 up-regulated and down-regulated GO:BP terms for each species and by media, by calculating the average of the relative expression of each term for the 2 strains. Because of the low number of DEGs for *E. faecalis*, only 11 terms were consistently down-regulated in BHIg.

Figure S5. Addition of IMP restores biofilm formation in *de novo* purine mutants. Biofilms of *S. aureus* USA300 containing transposon insertion (NTML) in gene encoding for the purine biosynthesis pathway were formed in TSBg, with

or without IMP (1 mg/mL) and quantified by crystal violet. Each bar represents biofilm production (in OD_{590}) of 18 replicates ± SEM (Student t-test; * P-value < 0.05; ** P-value < 0.01).

Figure S6. Inhibition of *de novo* purine biosynthesis pathway affects biofilm formation. Biofilms of *S. aureus* USA300 containing transposon insertion (NTML) in gene encoding for the purine biosynthesis pathway were formed in TSBg for 24 h, and examined by confocal microscopy. Biofilms were stained with Concanavalin A Alexa Fluor™ 647 conjugate (pink) for exopolysaccharides, and Syto9 (green) for cells. Images are representative of 2 biological replicates, and scale bar is 10 µm.

Table S1. Strains used in this study

Table S2. Prokaryotic Illumina libraries primers.

Table S3. DEGS commonly regulated in all conditions for the two strains of the same species.

Table S4. DEGs from the media influence transcriptomic analysis.

Table S5. Core genome analysis of *S. aureus* and *E. faecalis* strains with PATRIC Proteome analysis services.

Table S6. Abbreviations used in the purine biosynthesis pathway.
